# Exciton–phonon coupling strength in single-layer MoSe_2_ at room temperature

**DOI:** 10.1038/s41467-021-20895-0

**Published:** 2021-02-11

**Authors:** Donghai Li, Chiara Trovatello, Stefano Dal Conte, Matthias Nuß, Giancarlo Soavi, Gang Wang, Andrea C. Ferrari, Giulio Cerullo, Tobias Brixner

**Affiliations:** 1 Institut für Physikalische und Theoretische Chemie, Universität Würzburg, Am Hubland, 97074 Würzburg, Germany; 2 Dipartimento di Fisica, Politecnico di Milano, Piazza L. da Vinci 32, I-20133 Milano, Italy; 3 Cambridge Graphene Centre, University of Cambridge, 9 JJ Thomson Avenue, Cambridge, CB3 0FA UK; 4 Institute for Solid State Physics, Abbe Center of Photonics, Friedrich-Schiller-University Jena, Max-Wien-Platz 1, 07743 Jena, Germany; 5 IFN-CNR, Piazza L. da Vinci 32, I-20133 Milano, Italy; 6 Center for Nanosystems Chemistry (CNC), Universität Würzburg, Theodor-Boveri-Weg, 97074 Würzburg, Germany

**Keywords:** Two-dimensional materials, Fluorescence spectroscopy

## Abstract

Single-layer transition metal dichalcogenides are at the center of an ever increasing research effort both in terms of fundamental physics and applications. Exciton–phonon coupling plays a key role in determining the (opto)electronic properties of these materials. However, the exciton–phonon coupling strength has not been measured at room temperature. Here, we use two-dimensional micro-spectroscopy to determine exciton–phonon coupling of single-layer MoSe_2_. We detect beating signals as a function of waiting time induced by the coupling between A excitons and *A*′_1_ optical phonons. Analysis of beating maps combined with simulations provides the exciton–phonon coupling. We get a Huang–Rhys factor ~1, larger than in most other inorganic semiconductor nanostructures. Our technique offers a unique tool to measure exciton–phonon coupling also in other heterogeneous semiconducting systems, with a spatial resolution ~260 nm, and provides design-relevant parameters for the development of optoelectronic devices.

## Abstract (web summary)

The exciton–phonon coupling (EXPC) affects the opto-electronic properties of atomically thin semiconductors. Here, the authors develop two-dimensional micro-spectroscopy to determine the EXPC of monolayer MoSe_2_.

## Introduction

Layered materials (LMs)^1–4^, such as single-layer (1L) transition metal dichalcogenides (1L-TMDs)^5–8^, are a promising platform for new photonic and optoelectronic devices. Bulk semiconducting TMDs consist of covalently bound layers of type MX_2_, where M is a metal (e.g., Mo, W) and X is a chalcogen atom (e.g., S, Se), held together by van der Waals interactions^3^. When they are exfoliated, or grown as 1L, quantum confinement induces an indirect-to-direct bandgap transition^5,6^. The reduced dimensionality is also responsible for high exciton binding energies (hundreds of meV)^7,8^, making 1L-TMDs excellent candidates for optoelectronic devices at room temperature (RT)^2^.

Exciton–phonon coupling (EXPC) plays a key role in determining the temperature-dependent optoelectronic and transport properties of 1L-TMDs^9–11^. It is responsible for, e.g., non-radiative exciton decay^9,10,12^, limiting the fluorescence quantum yield^11^, the formation of dark-exciton phonon replicas^13^, and it mediates spin-flip processes, thus decreasing the lifetime of spin/valley-polarized charge carriers^14^. For temperature < 100 K, the interaction between excitons and acoustic phonons induces linewidth broadening and dominates the excitonic resonances of 1L-TMDs^9,15,16^. The situation is different for higher temperature. Ref. ^17^ suggested that the coupling between excitons and optical phonons induces sidebands in the absorption spectrum of 1L-MoSe_2_ at RT. Yet the spectral signature of this coupling is obscured by inhomogeneous broadening^17^. The presence of EXPC was inferred from resonant Raman scattering^18,19^, as well as time-resolved transmission measurements^20,21^, where the *A*′_1_ optical phonon mode was observed to couple with the A excitonic resonance. While the exciton energies can be obtained from photoluminescence (PL) and those of (ground state) phonons from Raman measurements, this does not fully characterize the system. To obtain the complete Hamiltonian, one also requires the displacement along the phonon coordinate of the exciton-state potential energy minimum versus the ground state. This displacement is the EXPC strength, and determines how strongly phonons are excited upon an optical transition to the exciton state. To the best of our knowledge, the EXPC strength was not measured for any 1L-TMDs at RT, because overtone bands of the optical phonon mode were not detectable^18–21^. We determine this missing quantity in the present work.

Optical four-wave-mixing experiments in semiconductors provide access to coherent dynamics of excitons^10,22–24^. In photon echo experiments the polarization state of incident photons (circular or linear) allows one to uncover different mechanisms behind the signal formation^25,26^. Different levels can be distinguished by the polarization dependence^26–28^. Two-dimensional electronic spectroscopy (2DES) is a powerful tool to analyze light-induced coherences in molecular systems^29–32^ and semiconductors^33,34^. It is a generalized version of transient absorption spectroscopy, providing frequency resolution not only for the probe step, but also for the pump^35–39^. Coherent broadband excitation of several quantum energy levels leads to wave packets that may be detected as oscillations of specific peaks in the 2d maps as a function of waiting time *T*^30,40^. Analysis of frequency, decay time, and the position of such oscillations allows one to explore the underlying energy structure and the coupling mechanism leading to level splittings^30,40,41^. Ref. ^41^ theoretically proposed that an additional Fourier transform along *T* and cutting the resulting 3d spectrum at certain beating frequencies could lead to 2d maps that are sensitive to the EXPC strength.

It is challenging to apply 2DES on micro-scale samples or heterogeneous materials with localized structural domains on a μm lateral scale, because the standard phase-matching geometry requires the exciting beams to be non-collinear with respect to each other^37^. This cannot be realized simultaneously when focusing with a high-numerical-aperture (NA) objective, in which all incident light arrives at the sample from the same solid angle. As a result, if one chooses to employ phase matching, this necessarily requires longer focal lengths, leading to larger spot sizes and unwanted averaging over different spatial regions or crystal orientations^42^. Instead, one can also select the signal by phase cycling^43–45^, which relies on detecting population-based signals as a function of inter-pulse phase combinations^43,45,46^. The collinear geometry accessible with phase cycling enables 2d micro-spectroscopy, i.e., the combination of 2DES with fluorescence microscopy, to gain additional spatial resolution^42,47^.

Here, we develop 2d micro-spectroscopy to resolve the spectral features of the phonon sidebands in 1L-MoSe_2_ at RT and determine the EXPC. We observe oscillations in 2d maps that arise from the coupling between the *A*′_1_ optical phonon mode and the A exciton. From comparison with simulated 2d beating maps, we deduce a Huang–Rhys factor *S* ~ 1. This implies a large EXPC strength for 1L-MoSe_2_, when compared with other inorganic semiconductor nanostructures, such as CdSe quantum dots^48^ and rods^49^, ZnSe quantum dots^50^, and single-wall carbon nanotubes^51^, most of which fall in the range ~ 0–0.5^52^, providing design-relevant information for the development of photonic devices based on 1L-MoSe_2_. Our method can be extended to other 1L-TMDs and LMs and to other important semiconducting systems, for which the ~260-nm spatial resolution of micro-spectroscopy is required, e.g., single-wall carbon nanotubes, LM heterostructures, layered perovskites, bulk heterojunctions, or microcavities with embedded semiconductors.

## Results and discussion

**Fig. 1:**
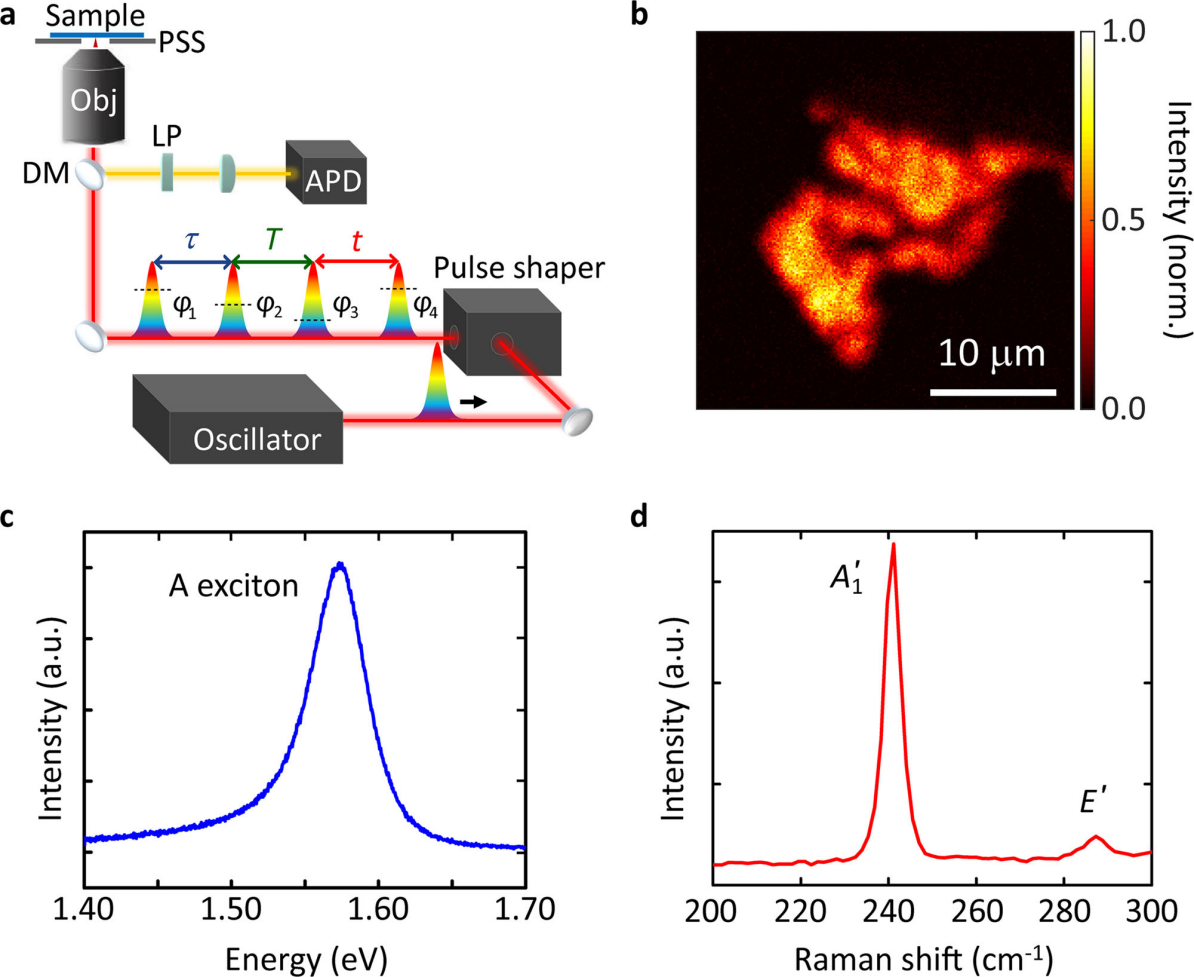
Overview of setup and the sample. **a** Fluorescence-detected 2d micro-spectroscopy setup. Four collinear laser pulses are generated by a pulse shaper with controllable inter-pulse time delays (*τ*, *T*, *t*) and phases (*φ*_*i*_, *i* = 1, 2, 3, 4) and focused by a high-NA objective (Obj). The position of the sample is controlled by a piezo scanning stage (PSS). The dichroic mirror (DM) under the objective transmits the excitation beam (red) and reflects the PL signal (yellow). A long-pass filter (LP) is used to block the remaining excitation beam. The PL signal is detected by an avalanche photodiode (APD). **b** PL map obtained with the setup of panel **a**. **c** PL and **d** Raman spectrum for 514 nm excitation. The peak in the PL spectrum corresponds to the A exciton. The Raman spectrum shows the out-of-plane *A*′_1_ mode ~ 241 cm^−1^, and the in-plane *E*′ mode ~ 288 cm^−1^.

The experimental setup is sketched in Fig. 1a. A Ti:sapphire oscillator emits 12-fs pulses at 80 MHz repetition rate. A pulse shaper generates a collinear four-pulse sequence, focused by a high-NA = 1.4 objective, so that a spatial resolution ~ 260 nm is achieved. To image the sample, the laser focus is mapped by a piezo scanning stage, and the PL signal is detected by an avalanche photodiode (APD). For the 2d map, the PL intensity is detected while scanning a first coherence time *τ* (delay between the first two pulses), a waiting time *T* (delay between second and third pulse), and a second coherence time *t* (delay between third and fourth pulse, Fig. 1a). Fourier transformation over *τ* and *t* results in a 2d map for every *T* (see Methods for data acquisition details). Nonlinear signals are obtained by systematically scanning through a number of discrete phase steps for each pulse and for each pulse-delay combination. Rephasing and nonrephasing signals are retrieved as linear superpositions of differently phase-modulated data^45^.

We investigate mechanically exfoliated 1L-MoSe_2_ on a 200-μm fused silica substrate (see Methods for details). Figure 1b is a PL map, taken with the setup of Fig. 1a, for a representative sample. 1L-MoSe_2_ has a direct bandgap at the K point of the Brillouin zone leading to two excitonic transitions A and B ~1.57 and 1.75 eV^53^. The PL spectrum (Fig. 1c) shows a single peak ~1.57 eV, due to the radiative recombination of A excitons^54^. The signal of the trion is much weaker than that of the neutral exciton at RT^24,55^. In our experiment we detect predominantly the neutral exciton. This is confirmed by the linear PL spectrum of our sample (Fig. 1c), in which the main peak is located at a position that agrees with that found for neutral excitons^54^. The Raman spectrum measured at 514 nm (Fig. 1d) shows the out-of-plane *A*′_1_ mode ~241 cm^−1^ with full width at half maximum (FWHM) ~ 4 cm^−1^, and the in-plane *E*′ mode ~288 cm^−1^ (FWHM ~ 6 cm^−1^). Both PL and Raman spectra confirm that the sample is 1L-MoSe_2_^18,54^.

**Fig. 2:**
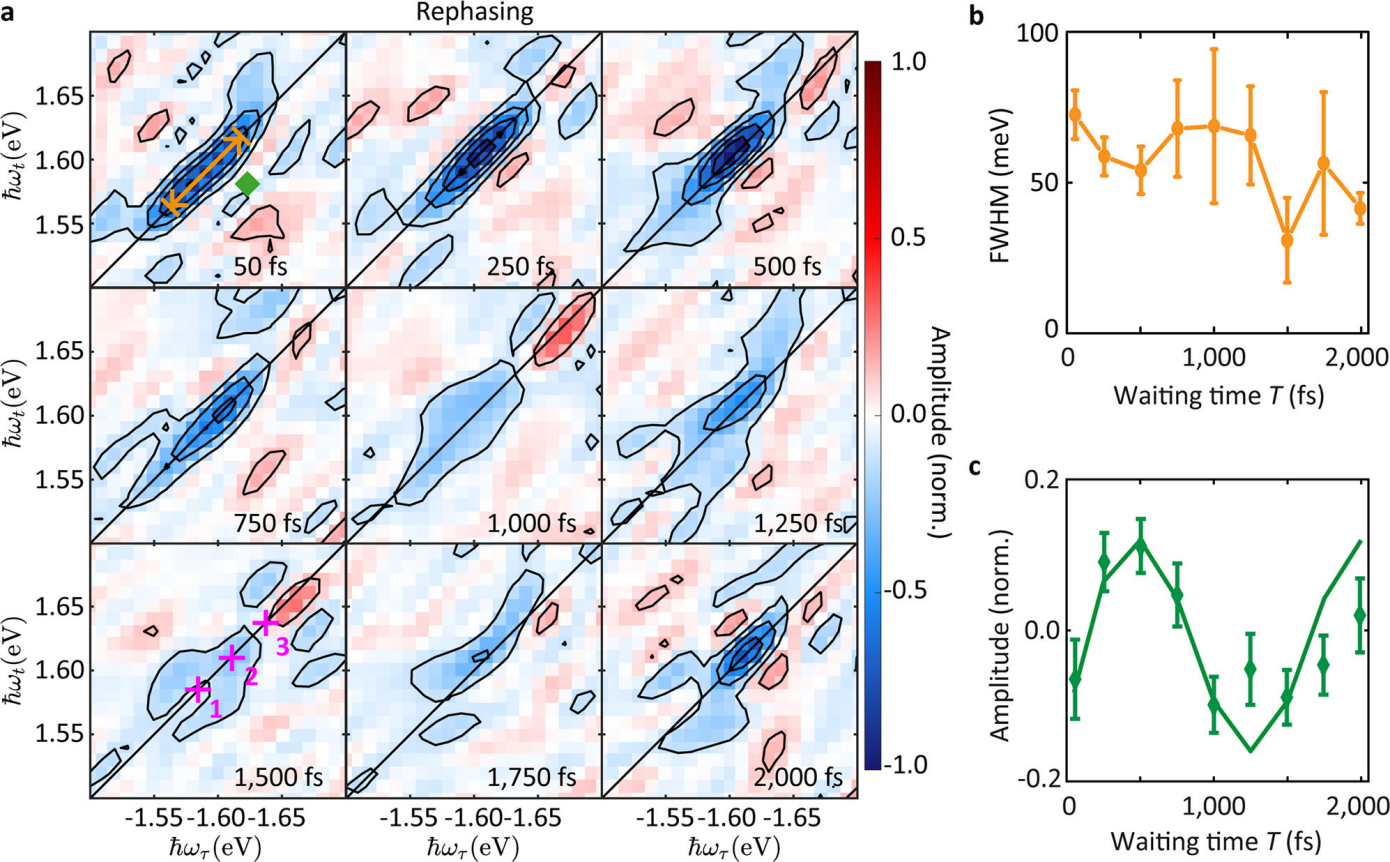
Beating signal in the rephasing 2d maps. **a** Rephasing 2d maps at different *T*, normalized to the maximum absolute value of the real part of the map at *T* = 500 fs. **b** Diagonal linewidth (FWHM, indicated by the orange double arrow at *T* = 50 fs in panel **a**) versus *T*. The error bars depict 95% confidence bounds from fitting the diagonal slices by a Gaussian function. **c** Amplitude evolution (green diamonds) of one pixel (marked by the green diamond at *T* = 50 fs in panel **a**) and fit (solid green curve). The error bars are evaluated by calculating the fluctuations within a region containing background noise (see Supplementary Note 4).

The rephasing 2d maps in the region around the A exciton are shown in Fig. 2a for various *T*, while the nonrephasing and absorptive 2d maps are in Supplementary Figs. 1 and 5, respectively. The peak linewidth along the diagonal direction of the rephasing map (orange double arrow in upper left panel) is plotted versus *T* in Fig. 2b. Closer analysis of the systematic variation of this linewidth with *T* (Supplementary Note 2) indicates that there are three components along the diagonal, marked with purple crosses in the lower left panel of Fig. 2a, whose amplitudes oscillate, but not in phase. Thus, when *T* ~ 1500 fs, the amplitude of the middle component is much higher than the other two, minimizing the effective diagonal linewidth (minimum in Fig. 2b). The measured 2d maps capture the fourth-order nonlinear optical response, as sixth-order contributions are negligible (see Supplementary Note 3).

We then extract the amplitude evolution of a typical pixel (marked by the green diamond in the 2d map at 50 fs) as a function of *T* (Fig. 2c). The number of points is restricted, due to the long measurement time (26 h for one point). A long-lived (>2 ps) oscillation with amplitude above the noise level is observed. The reproducibility of the data is confirmed by a second measurement for the same *T* in Supplementary Note 4.

We now analyze the origin of the oscillations in the 2d maps with the goal to deduce the EXPC strength. Previous experiments reported that the trion signal in 1L-MoSe_2_, located ~0.03 eV below the neutral exciton peak^55^, gradually dies out both in PL and absorption, when the temperature increases from 15 to 295 K, while the signal intensity of neutral excitons remains nearly unchanged^24,55^. Thus, the signal of the trion is much weaker than the neutral exciton at RT and in our experiment we detect predominantly the neutral exciton. This implies that wave packets involving trions can be excluded as a source of the long-lived (>2 ps) RT oscillations in Fig. 2c. Biexciton signals can be excluded in our 2d measurements due to their thermal dissociation at RT and cancellation of excited-state absorption pathways in fluorescence-detected 2d spectroscopy (see Supplementary Note 5). Vibrational wave packets were reported at RT in Refs. ^20,21^, with a dephasing time ~4.5 ps for 1L- and few-layer WSe_2_^20^ and ~1.7 ps for 1L-MoS_2_^21^. Therefore, EXPC can explain the oscillations in our 2d maps. We extract the phonon energy from a fit (Fig. 2c, solid green curve) and obtain, even for our undersampled data (less than one data point for each oscillation period as a result of a compromise arising from finite available data acquisition time), an oscillation period ~136 ± 2 fs (see Supplementary Note 6 for the fitting procedure). This corresponds to an energy splitting between the participating states ~30.4 ± 0.4 meV, matching the optical *A*′_1_ phonon mode’s energy ~29.9 meV, i.e., 241 cm^−1^, as measured in the Raman spectrum of Fig. 1d.

**Fig. 3:**
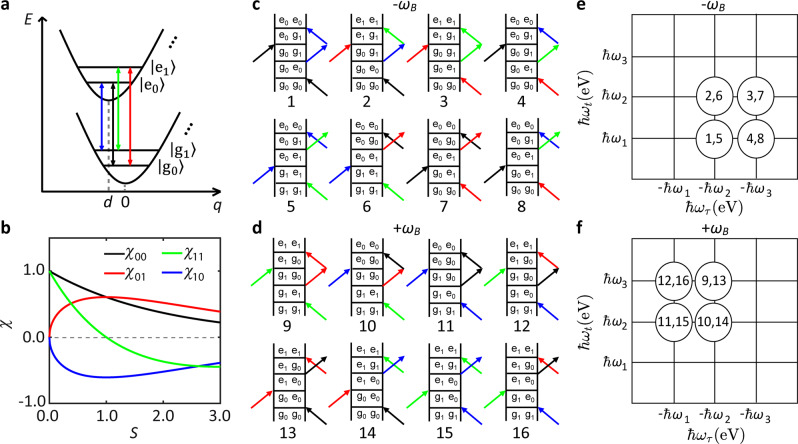
Analysis of beating signals. **a** Schematic diagram of displaced harmonic oscillators (Franck–Condon coupling model^56^) with two vibrational levels ($\left| {{\mathrm{g}}_0} \right\rangle$, $\left| {{\mathrm{g}}_1} \right\rangle$) in the electronic ground state, and two in the electronic excited state ($\left| {{\mathrm{e}}_0} \right\rangle$, $\left| {{\mathrm{e}}_1} \right\rangle$). The horizontal shift between the two potential minima, *d*, characterizes the EXPC strength. Transitions $\left| {{\mathrm{g}}_0} \right\rangle$–$\left| {{\mathrm{e}}_0} \right\rangle$, $\left| {{\mathrm{g}}_0} \right\rangle$–$\left| {{\mathrm{e}}_1} \right\rangle$, $\left| {{\mathrm{g}}_1} \right\rangle$–$\left| {{\mathrm{e}}_0} \right\rangle$, and $\left| {{\mathrm{g}}_1} \right\rangle$–$\left| {{\mathrm{e}}_1} \right\rangle$ are color-coded in black, red, blue, and green, respectively. **b** Dependencies of Franck–Condon amplitudes *χ*_*ij*_ (*i*, *j* = 0 or 1) on *S*, which scales, d^2^/2. **c**, **d** Feynman pathways giving rise to the beating signals with **c** negative beating frequency −*ω*_*B*_, and, **d** positive frequency +*ω*_*B*_. **e** Beating-map locations of numbered Feynman pathways from panel **c**. **f** Beating-map locations of numbered Feynman pathways from panel **d**.

We define the EXPC strength using the Huang–Rhys factor, *S*, in the framework of the Franck–Condon coupling model^56^ (see Supplementary Note 7 for a definition of *S*), with the minimum number of states needed to describe the observed data (Fig. 3a). The model of Fig. 3a delivers 3 transition energies, as observed experimentally (purple crosses in Fig. 2a). We assign component 1 (with the lowest energy $\hbar \omega _1$) to the transition between $\left| {{\mathrm{g}}_1} \right\rangle$ and $\left| {{\mathrm{e}}_0} \right\rangle$ (blue in Fig. 3), component 2 (with a higher energy $\hbar \omega _2$) to the two degenerate transitions between $\left| {{\mathrm{g}}_0} \right\rangle$ and $\left| {{\mathrm{e}}_0} \right\rangle$ and between $\left| {{\mathrm{g}}_1} \right\rangle$ and $\left| {{\mathrm{e}}_1} \right\rangle$ (black and green, respectively), and component 3 (with the highest energy $\hbar \omega _3$) to the transition between $\left| {{\mathrm{g}}_0} \right\rangle$ and $\left| {{\mathrm{e}}_1} \right\rangle$ (red).

Transitions between $\left| {{\mathrm{g}}_0} \right\rangle$ and $\left| {{\mathrm{e}}_{i \ge 2}} \right\rangle$ states are not observed in the 2d maps. This agrees with resonance Raman scattering^18,19^ and their time-domain analogs^20,21^, where the *A*′_1_ overtone was not detected. This may imply an efficient non-radiative decay channel for the $\left| {{\mathrm{e}}_2} \right\rangle$ state, which results in a fast dephasing time for the hot vibronic band transitions. Transitions between $\left| {{\mathrm{g}}_{i \ge 2}} \right\rangle$ and $\left| {{\mathrm{e}}_0} \right\rangle$ are also not observed in the 2d maps, which can be explained as a negligible thermal population of $\left| {{\mathrm{g}}_{i \ge 2}} \right\rangle$ due to a small Boltzmann factor at RT. The transition amplitudes between different vibronic sublevels (blue, black, green, and red arrows in Fig. 3a) are proportional to the overlap of the vibrational wave functions of initial and final state, i.e., the Franck–Condon amplitudes *χ*^57^, plotted as a function of *S* in Fig. 3b. At *S* = 0, the red and blue curves are zero, indicating that it is not possible to excite $\left| {{\mathrm{e}}_1} \right\rangle$ starting from $\left| {{\mathrm{g}}_0} \right\rangle$ or to reach $\left| {{\mathrm{g}}_1} \right\rangle$ from $\left| {{\mathrm{e}}_0} \right\rangle$, thus the electronic/excitonic excitation is decoupled from vibrations.

We now correlate *S* with the oscillatory signals. We perform an additional Fourier transformation of the 2d maps with respect to *T*. This gives rise to a 3d spectrum, which is a hypercube as a function of $\hbar$*ω*_*τ*_, $\hbar$*ω*_*T*_ and $\hbar$*ω*_*t*_. 2d cuts at $\hbar \omega _T$ =$\,+ \hbar \omega _B$ and $\hbar \omega _T$ =$\,- \hbar \omega _B$ result in two 2d beating maps, where *ω*_*B*_ is the beating frequency induced by EXPC.

Figure 3c lists all possible rephasing Feynman pathways that can result in contributions at negative beating frequency −*ω*_*B*_. Their individual positions in the 2d beating maps are in Fig. 3e. Figure 3d contains the contributions at positive *ω*_*B*_, and Fig. 3f their positions in the 2d map. The determination of all peak positions of individual Feynman pathways in 2d beating maps is introduced in Supplementary Note 8. Adding all pathways, we expect the beating map to be located on the lower right of the diagonal for negative beating frequency (Fig. 3e), and on the upper left for positive (Fig. 3f). The precise shape of the overall beating map depends on the relative amplitudes of the individual Feynman pathways. Those depend on the initial populations of $\left| {{\mathrm{g}}_0} \right\rangle$ and $\left| {{\mathrm{g}}_1} \right\rangle$, hence on the sample temperature, and on the products of the Franck–Condon amplitudes of the four involved transitions (colored arrows in Fig. 3c, d) that in turn depend on *S* (Fig. 3b). Thus, analyzing the shape of the beating maps allows us to estimate *S*.

**Fig. 4:**
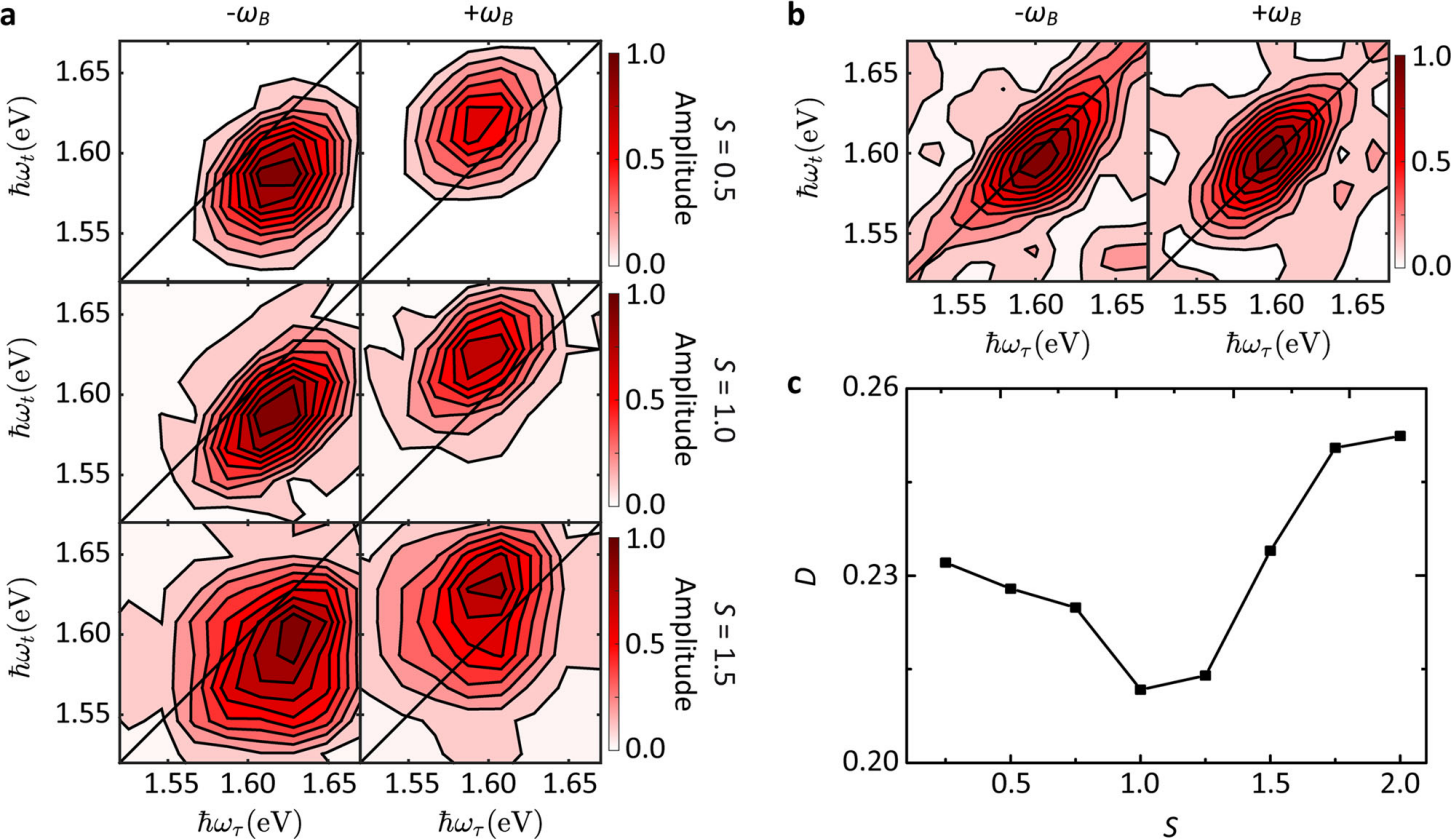
2d beating maps. **a** Simulated 2d beating maps for –*ω*_*B*_ (left) and +*ω*_*B*_ (right) and *S* = 0.5, 1, 1.5 from top to bottom rows. **b** Measured 2d beating maps with −*ω*_*B*_ (left) and +*ω*_*B*_ (right). **c** *D* between measured and simulated 2d beating maps versus *S* used in the simulation.

For a quantitative evaluation, we simulate the 2d beating maps by numerically solving a Lindblad master equation^58^ for a system described by the Franck–Condon model illustrated in Fig. 3a (see Methods for details). *S* is varied from 0.25 to 2 with a step size of 0.25. Figure 4a plots the simulation for *S* = 0.5, 1, 1.5 from top to bottom. Data for other *S* are in Supplementary Fig. 13. We recognize the expected features of Fig. 3e, f. The pathway contributions overlap with each other, due to line broadening along the diagonal and anti-diagonal directions. For *S* = 1.5, the four underlying subpeaks create a square lineshape. For smaller *S*, the anti-diagonal linewidth changes strongly because of the varying relative contributions of the different Feynman pathways, leading to one asymmetric peak in each 2d beating map, whose center is located below (above) the diagonal line for negative (positive) beating frequency as predicted in Fig. 3e, f. The change in linewidth can be understood by considering that *χ*_11_ (Fig. 3b, solid green curve) crosses zero (the dashed gray line) for *S* = 1, such that only Feynman pathways 1, 7, 11, 13, e.g., without $\left| {{\mathrm{g}}_1} \right\rangle$–$\left| {{\mathrm{e}}_1} \right\rangle$ transition (green arrow in Fig. 3c, d), contribute. Therefore, the anti-diagonal linewidth reaches a minimum for *S* = 1.

Figure 4b shows the experimental 2d beating maps at –*ω*_*B*_ (left) and +*ω*_*B*_ (right), obtained as cuts through the rephasing 3d spectrum at the same beating frequency as in the simulations, *ω*_*B*_ = 4.6 × 10^13^ s^−1^. The asymmetry with respect to the diagonal is visible, and the elliptical shape [rather than roundish (small *S*) or squarish (large *S*)] points at an intermediate *S* by comparison with simulations. The lowest contour lines of experimental and simulated beating maps in Fig. 4a, b show some “jagged” behavior. The factors that could contribute to this are discussed in Supplementary Note 9.

**Fig. 5:**
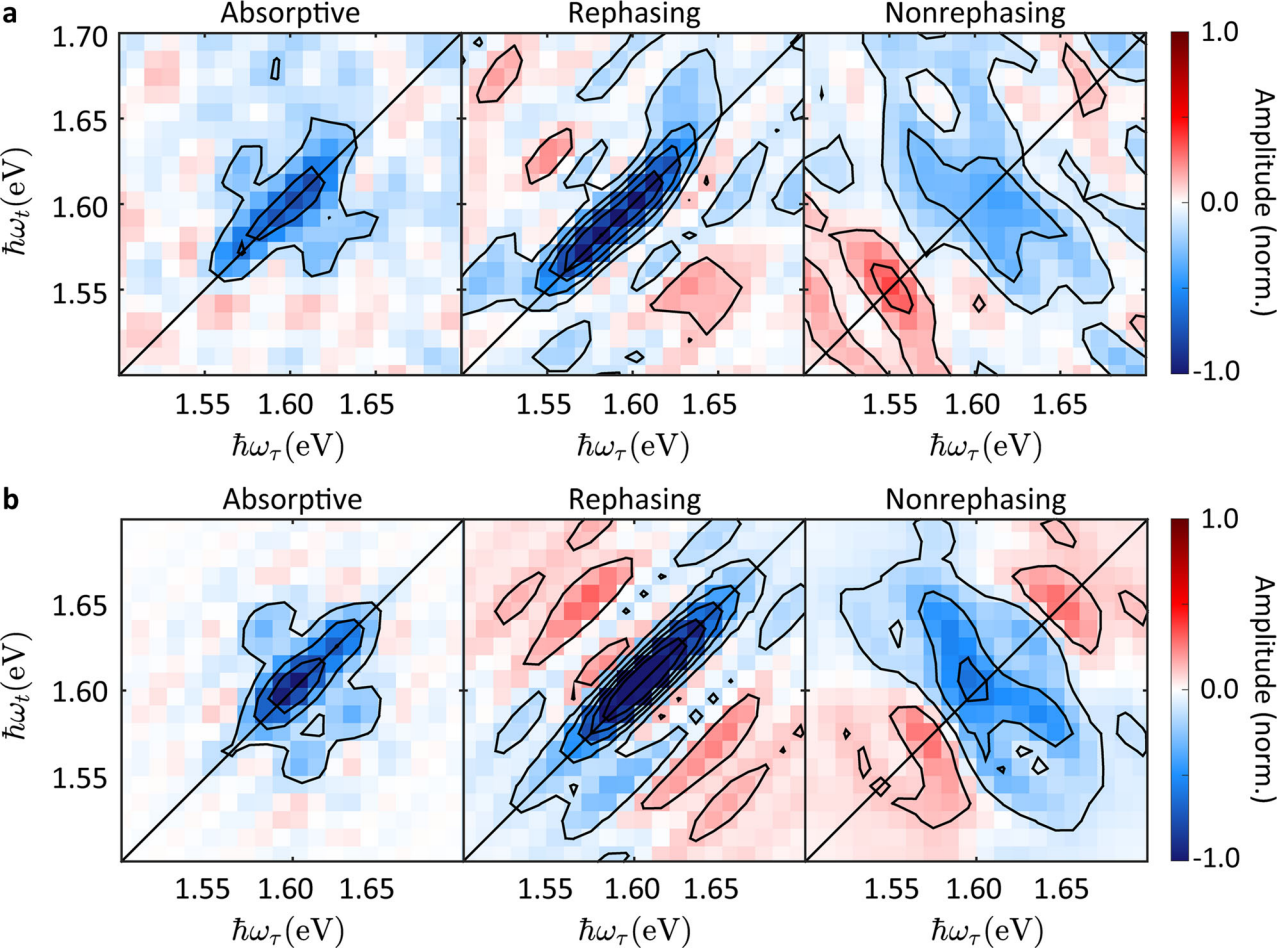
Absorptive (left), rephasing (middle), and nonrephasing (right) real-valued 2d maps at *T* = 50 fs. **a** Experiment. **b** Simulation using the deduced optimal *S* = 1.

To determine the EXPC strength quantitatively, we calculate the deviation *D* between measured and simulated 2d beating maps:

1 $$D = \sqrt {\frac{1}{{N^2}}\mathop {\sum }\limits_{i = 1}^N \mathop {\sum }\limits_{j = 1}^N \left( {A_{ij} - \tilde A_{ij}} \right)^2}$$

where *N* is the pixel number in each dimension of the 2d beating maps, *A*_*ij*_ ($\tilde A_{ij}$) is the amplitude of the pixel in column *i* and row *j* of the simulated (experimental) 2d beating map. Figure 4c plots *D* versus *S*. We find the best agreement for *S* = 1. We then compare the experimental absorptive, rephasing, and nonrephasing 2d maps for *T* = 50 fs (Fig. 5a) with the simulation using the optimal *S* (Fig. 5b) and find good agreement, confirming the reliability of our Franck–Condon model.

We note that large *S* on the order of 1 in 1L-TMDs are supported by theory^59–61^, but were never previously experimentally measured, to the best of our knowledge. The exciton coupling with longitudinal optical phonons in 1L-TMDs was studied by ab initio calculations^59,60^. These found that polar LO phonon vibrations give rise to a macroscopic electric field that couples to the charge carriers. Such a coupling, named “Fröhlich interaction”, is fundamentally affected by the dimensionality of the system. When the dimensionality of the system decreases from 3d to 2d, a 3-fold increase of *S* is predicted, see, e.g., Fig. 7 in Ref. ^62^. Taking into account Fröhlich interactions in a 2d model, Ref. ^61^ calculated *S* for LO phonons as a function of the polarization parameter for 1L-MoSe_2_, finding ~1.93–2.24. Defects may also have a strong influence on *S*^63,64^. The electric fields induced by local charges at interfaces increase the non-vanishing part of the electron and hole polaron clouds in the exciton state^64^ and, as a result, *S* as large as ~1 can be found^64^.

In conclusion, we carried out spatially resolved, fluorescence-detected 2d micro-spectroscopy on 1L-MoSe_2_. We identified phonon sidebands upon excitation of the A exciton, due to coupling to the optical phonon mode *A*′_1_. While the phonon is not resolved in linear absorption or PL spectra at RT, analysis of the 2d beating frequency as a function of waiting time allowed us to assign the phonon mode via comparison with Raman data. We determined the exciton–phonon coupling strength, i.e., the displacement along the phonon coordinate of the excited-exciton oscillator potential with respect to the ground state, and found a Huang–Rhys factor, *S* ~ 1, by comparison with simulations of 2d beating maps. The measured *S* ~ 1 is larger than most reported values (*S* ~ 0–0.5) of other inorganic semiconductor nanostructures^52^, such as CdSe quantum dots^48^ and rods^49^, ZnSe quantum dots^50^, single-wall carbon nanotubes^51^, indicating a strong EXPC. This finding may benefit, amongst others, the development of TMD-based polariton devices^65^, in which the polariton-relaxation process strongly depends on the EXPC strength^66^.

Our space-, time-, and excitation/detection-frequency-resolved spectroscopy provides a unique tool to measure EXPC also in other TMDs. hBN encapsulation can lower inhomogeneous broadening of 1L-TMDs^67,68^, we thus expect better resolved peaks for hBN-encapsulated samples. *S* may be influenced by the substrate, by changing the macroscopic electric field induced by the polar LO phonon at the interface^60^. E.g., SiO_2_ increases the screening of the Fröhlich interaction strongly at small momenta^60^. Therefore, we expect that a different substrate might result in a different *S*, hence, hBN encapsulation might also influence it. Our method can be extended to other semiconducting systems for which phonon-induced subbands are expected in the excitonic lineshape, such as single-wall carbon nanotubes^69^, layered perovskites^70^, bulk heterojunctions^71^, or other organic crystals. Because of the high spatial resolution ~260 nm, our technique can also be used to study excitonic coupling in layered materials heterostructures or microcavities with embedded semiconductors. The determination of EXPC will provide design-relevant parameters for the development of photonic and optoelectronic devices based on these semiconducting systems.

## Methods

### Samples fabrication

The samples are prepared by micromechanical cleavage^72^ of bulk MoSe_2_ from HQ Graphene. This is performed with polydimethylsiloxane (PDMS) and, after inspection under an optical microscope, 1L-MoSe_2_ is dry transferred in ambient conditions to a 200-μm fused silica substrate^73^. After transfer, the samples are characterized by Raman and PL with a Renishaw Invia spectrometer at 514 nm and with a ×50 objective. Metallic frames (Cr/Au) are fabricated around selected 1L-MoSe_2_ flakes on fused silica by laser-writer lithography to facilitate the identification of the samples’ position for subsequent 2DES characterization.

### Data acquisition

A femtosecond oscillator (Venteon Laser Technologies GmbH, Pulse One PE) provides a laser spectrum ranging from 650 to 950 nm, confined by a hard aperture in the Fourier plane of a 4*f*-based pulse shaper in front of the liquid-crystal display (LCD, Jenoptik Optical Systems GmbH, SLM-S640d). The aperture acts as a short-pass filter at 808 nm, so that the longer-wavelength PL can be detected without scattering from the pump light. A Schott KG5 color filter further modulates the spectrum into a smooth shape, which ensures the absence of pronounced side peaks and other irregularities in the temporal pulse profile. The laser focus in the microscope is mapped by a piezo scanning stage (P-517.3CL, PI, Germany). Excitation occurs through a focusing objective (Nikon Plan Apo, 100×/1.40). PL is collected through the same objective, transmitted through a dichroic beam splitter (DBS, AHF Analysentechnik, F48-810) and an additional emission filter (EF, AHF Analysentechnik, F76-810), and detected by an APD (Perkin Elmer, SPCM-CD 2801).

We compress the laser pulses by (1) using chirped mirrors to pre-compensate some second-order phase dispersion; (2) employing the pulse shaper to compensate any remaining dispersion. A two-photon photodiode (TPPD) is placed in the focus of the microscope objective to generate a nonlinear feedback signal that is a measure of pulse intensity and pulse duration. We then utilize the algorithm of Ref. ^74^ to maximize the peak intensity, leading to a transform-limited laser pulse. To characterize the result of pulse compression, an autocorrelation trace is measured using the same TPPD, as shown in Supplementary Fig. 15. This agrees well with a simulated one assuming the experimentally measured laser spectrum and a flat spectral phase. This correspondence indicates successful phase-dispersion compensation and ~12 fs pulses at the sample position, as discussed in Refs. ^47,74^.

Linearly polarized light, acting as a superposition of left- and right-handed circularly polarized light, is used to simultaneously excite both the transitions in the K and K’ valleys. The pump fluence is ~2 μJ/cm^2^. We estimate the heating through laser irradiation during the experiment as discussed in Supplementary Note 10. The sample temperature increases from 300 to ~308 K within the first 100 ns, then remains constant. Thus there is no unwanted heating, thermal instabilities or damage.

We obtain the 2d maps by scanning *τ* and *t* in steps of 3 fs each from 0 to 99 fs, for *T* = 50, 250, 500, 750, 1000, 1250, 1500, 1750, 2000 fs, using the spectral modulation function^75^:

2 $$M\left( \omega \right) =	\, {\mathrm{exp}}\left[ {i\left( {\omega - \omega _0\left( {1 - \gamma } \right)} \right)\left( { - \tau - T} \right)} \right] + {\mathrm{exp}}\left[ {i\left( {\omega - \omega _0\left( {1 - \gamma } \right)} \right)\left( { - T} \right) + i\varphi _{12}} \right]\\ 	+ {\mathrm{exp}}\left[ {i\varphi _{13}} \right] + {\mathrm{exp}}\left[ {i\left( {\omega - \omega _0\left( {1 - \gamma } \right)} \right)t + i\varphi _{14}} \right]$$

at a center frequency *ω*_*0*_ = 2.5 × 10^15^ s^−1^. We avoid undersampling with time steps of 3 fs by employing a partially rotating frame with *γ* = 0.2. The third pulse is fixed at time 0, so that when 2d maps are measured at a certain *T*, only the first and fourth pulses are delayed. By setting the phase of the first pulse to 0, three relative phases, i.e., *φ*_12_, *φ*_13_, and *φ*_14_, are scanned in a 27-step phase-cycling scheme, where each relative phase takes values of 0, $\frac{{2\pi }}{3}$, $\frac{{4\pi }}{3}$. This allows us to select rephasing and nonrephasing contributions individually from the complete raw data^47,76^. We obtain absorptive 2d maps by summing the real parts of the rephasing and nonrephasing 2d ones, canceling dispersion terms, leaving a pure absorptive lineshape^39^. Due to the finite response time of the liquid crystals of our pulse shaper, we wait ~500 ms after changing the phase mask before taking data. PL is averaged over ~1 ms for each APD acquisition period. Including additional averaging (2000 times for each pulse shape), the total measurement time for one 2d map is ~26 h. During the measurements the PL intensity of the sample is constantly monitored every ~80 s. We observe no systematic decay during the measurement time. This indicates a long-term chemical, thermal, and photostability of the sample. The group delay dispersion at the sample position is compensated by adding an additional phase to the modulation function^47^.

### Simulations

To simulate the 2d maps, we solve the Lindblad quantum master equation^58^

3 $$\frac{\partial }{{\partial t^{\prime} }}\rho \left( {t^{\prime} } \right) = - \frac{i}{\hbar }\left[ {{\cal{H}}\left( {t^{\prime} } \right),\rho \left( {t^{\prime} } \right)} \right] + \mathop {\sum }\limits_j \frac{1}{{T_j}}\left( {{\cal{L}}_j\rho \left( {t^{\prime} } \right){\cal{L}}_j^\dagger - \frac{1}{2}{\cal{L}}_j^\dagger {\cal{L}}_j\rho \left( {t^{\prime} } \right) - \frac{1}{2}\rho \left( {t^{\prime} } \right){\cal{L}}_j{\cal{L}}_j^\dagger } \right)$$

where the time evolution of the density matrix $\rho \left( {t^{\prime} } \right)$ of the quantum system under a Hamiltonian ${\cal{H}}\left( {t^{\prime} } \right)$ is treated in the Liouville–von Neumann formalism, with the extension of dissipative and pure dephasing effects, ${\cal{H}}\left( {t^{\prime} } \right)$ is expressed as the sum of a time-independent Hamiltonian ${\cal{H}}_0 = \hbar \omega _m\mathop {\sum}\nolimits_m {\left| m \right\rangle \left\langle m \right|}$ and an interaction Hamiltonian ${\cal{H}}_I\left( {t^{\prime} } \right) = \gamma _{{\mathrm{ex}}}E\left( {t^{\prime} } \right)\mathop {\sum}\nolimits_{m \ne n} {\mu _{m,n}\left( {\left| m \right\rangle \left\langle n \right| + \left| n \right\rangle \left\langle m \right|} \right)}$, where $\left| m \right\rangle$ (or $\left| n \right\rangle$) are the unperturbed eigenstates with eigenenergies $\hbar \omega _m$ (or $\hbar \omega _n$), *γ*_ex_ is the field coupling strength for excitation with external electric field $E\left( {t^{\prime} } \right)$, $\mu _{m,n}$ is the transition dipole moment between states $\left| m \right\rangle$ and $\left| n \right\rangle$, *T*_*j*_ represents the time associated with a pure dephasing or population relaxation process, and the Lindblad operators ${\cal{L}}_j$ are defined as ${\cal{L}}_j = a_n^\dagger a_n$ for pure dephasing and ${\cal{L}}_j = a_m^\dagger a_n$, with $m\, \ne\, n$ for a population relaxation process, where $a_m^\dagger$. and $a_n$ denote the creation and annihilation operators, respectively.

We assume a four-level system, with two vibrational levels in the ground electronic state ($| {{\mathrm{g}}_0} \rangle$ and $| {{\mathrm{g}}_1} \rangle$) and two vibronically excited states ($\left| {{\mathrm{e}}_0} \right\rangle$ and $\left| {{\mathrm{e}}_1} \right\rangle$), as in Fig. 3a. The splittings within the subbands are taken to be identical, i.e., we use the same energy separations (30 meV^20^) between $| {{\mathrm{g}}_0} \rangle$ and $| {{\mathrm{g}}_1} \rangle$ as well as between $\left| {{\mathrm{e}}_0} \right\rangle$ and $\left| {{\mathrm{e}}_1} \right\rangle$. The Franck–Condon amplitudes between $| {{\mathrm{g}}_i} \rangle$ and $| {{\mathrm{e}}_j} \rangle$, i.e., $\chi _{ij}$. (*i*, *j* = 0 or 1) depend on *S* as for Fig. 3b. The initial populations of $| {{\mathrm{g}}_0} \rangle$ and $\left. {{\mathrm{g}}_1} \right\rangle$ are determined by the temperature, according to the Boltzmann distribution. In Supplementary Note 10 we estimate the heating through laser irradiation during the experiment. We find the sample to remain close to RT.

The excitation laser field is calculated from the experimentally utilized laser spectrum assuming a flat phase and then adding the transfer function:

4 $$M\left( \omega \right) = 	\; {\mathrm{exp}}\left\{ {i\left[ {\omega - \omega _0\left( {1 - \gamma } \right)} \right] {T_{{\mathrm{off}}}}} \right\} + {\mathrm{exp}}\left\{ {i\left[ {\omega - \omega _0\left( {1 - \gamma } \right)} \right]\left( {T_{{\mathrm{off}}} + \tau } \right) + i\varphi _{12}} \right\} \\ 	+ {\mathrm{exp}}\left\{ {i\left[ {\omega - \omega _0\left( {1 - \gamma } \right)} \right]\left( {T_{{\mathrm{off}}} + \tau + T} \right) + i\varphi _{13}} \right\} \\ 	+ {\mathrm{exp}}\left\{ {i\left[ {\omega - \omega _0\left( {1 - \gamma } \right)} \right]\left( {T_{{\mathrm{off}}} + \tau + T + t} \right) + i\varphi _{14}} \right\}$$

where *T*_off_ is an offset of the position of the first pulse in time domain, set at 100 fs to avoid cutting off the first pulse at time zero. In the experimental modulation function of Eq. (2), time zero is set at the maximum of the third pulse, leading to a different mathematical expression. However, this difference does not affect the resulting 2d maps, since only relative time delays between the pulses are relevant. *τ* and *t* in the simulation are scanned with the same parameters as in the experiment, from 0 to 99 fs in steps of 3 fs with *γ* = 0.2, whereas *T* is scanned from 0 to 200 fs in steps of 25 fs.

Inhomogeneous broadening due to a Gaussian distribution of excitonic transition frequencies is taken into account by obtaining the inhomogeneously broadened response function, *S*_*I*_(*τ, t*), from the homogeneous response, *S*(*τ, t*), by solving Eq. (3), via:

5 $$S_I\left( {\tau ,t} \right) = S\left( {\tau ,t} \right) \cdot {\mathrm{exp}}\left[ { - \Delta ^2 \cdot \left( {\tau \mp t} \right)^2} \right]$$

where Δ is a parameter linearly proportional to the inhomogeneous linewidth broadening,—is applied for the rephasing signal, and + for the nonrephasing signal. Equation (5) is used under two assumptions. (1) Spectral diffusion can be ignored within the *T* = 2 ps window of the measurements. Typically, spectral diffusion is caused by environmental fluctuations around the transition dipoles, inducing a broadening along the anti-diagonal direction for the absorptive 2d maps as *T* increases^39^. This is not observed in our experiments (see Supplementary Fig. 5), indicating a much slower than 2 ps modulation time constant of the environment, justifying the use of Eq. (5). (2) The vibrational frequency does not change with the excitonic transition energy, also assumed for the model of Fig. 3a and Eqs. (1–3). If this was not fulfilled, a tilt of elongated peaks in the 2d beating maps relative to the diagonal would be observed^40^, unlike in our measurements (Fig. 4b).

## Supplementary information

Supplementary Information

## Data Availability

The data that support the findings of this study have been deposited in Mendeley Data with the embargo date of 1 March 2021. The data are available at the following link: 10.17632/52yj8d9p4t.1
