# Supplementary material for: Exciton–phonon coupling strength in single-layer MoSe2 at room temperature
Source: Nat Commun. 2021 Feb 11;12:954. doi: 10.1038/s41467-021-20895-0 (PMC7878916; doi:10.1038/s41467-021-20895-0)
Supplement: Supplementary file 1 — Supplementary Information [file 41467_2021_20895_MOESM1_ESM.pdf]

Supplementary Information:

Exciton–phonon coupling strength in single-layer $MoSe_2$ at room temperature

Donghai Li[1], Chiara Trovatello[2], Stefano Dal Conte[2], Matthias Nuß[1], Giancarlo Soavi,[3,4], Gang Wang[3], Andrea C. Ferrari[3], Giulio Cerullo[2,5*], and Tobias Brixner[1,6*]*

[1]Institut für Physikalische und Theoretische Chemie, Universität Würzburg, Am Hubland, 97074 Würzburg, Germany

[2]Dipartimento di Fisica, Politecnico di Milano, Piazza L. da Vinci 32, I-20133 Milano, Italy

[3]Cambridge Graphene Centre, University of Cambridge, 9 JJ Thomson Avenue, Cambridge CB3 0FA, UK

[4]Institute for Solid State Physics, Abbe Center of Photonics, Friedrich-Schiller-University Jena, Max-Wien-Platz 1, 07743, Jena, Germany

[5]IFN-CNR, Piazza L. da Vinci 32, I-20133 Milano, Italy

[6]Center for Nanosystems Chemistry (CNC), Universität Würzburg, Theodor-Boveri-Weg, 97074 Würzburg, Germany

*e-mails: acf26@eng.cam.ac.uk, giulio.cerullo@polimi.it, brixner@uni-wuerzburg.de

Supplementary Note 1: Rephasing and nonrephasing 2d maps

In collinear 2DES, rephasing and nonrephasing signals can be obtained separately by phase cycling[1–3]. The rephasing 2d maps of 1L-$MoSe_2$ at different T are in Fig. 2a in the main text, Supplementary Fig. 1 plots the nonrephasing 2d maps at different T. All the rephasing and nonrephasing 2d maps are normalized to the maximum absolute value of the real part of the rephasing maps at $T = 500$ fs.

In order to better understand the origin of the coherent oscillations in the measured 2d maps, we summarize some properties of double-sided Feynman diagrams[4]. We show exemplary Feynman diagrams for a three-level system (i.e., without vibrational sublevels within electronic states) of rephasing and nonrephasing pathways for population-detected 2DES (Supplementary Fig. 2, measuring fluorescence in collinear geometry) as well as for conventional coherence-detected 2DES (Supplementary Fig. 3, obtained in partially non-collinear geometry). The vertical lines represent time evolution of ket (left) and bra (right) states of the density matrix with time running from the bottom to the top. Every pulse induces an impulsive transition of the system within the density-matrix description[4], depicted by a solid arrow. An arrow pointing towards the quantum states in the middle increases the respective electronic quantum number, an arrow pointing away decreases it ($|g\rangle$ denotes the ground state, $|e\rangle$ the first excited state, and $|f\rangle$ the second excited state). Between two impulsive transitions, the system either remains in a population state (identical bra and ket states) or propagates in a coherent state (different bra and ket states)[4]. The sign of the signal of a specific Feynman pathway (labeled on the top of the Feynman diagram) depends on the number of interactions from the right side of the Feynman diagram, wherein each interaction leads to multiplication by -1 of the signal[4].

In all cases, the system is in a coherent state between the first and the second pulse and between the third and the fourth, i.e., the bra and ket states are different from each other. We call the associated time intervals excitation time (or first coherence time) τ and detection time (or second coherence time) t. Two-dimensional Fourier transformation with respect to these two coherence times results in a 2d map with the two axes of excitation frequency ω_τ (or energy $\hbar\omega_\tau$) and detection frequency ω_t (or energy $\hbar\omega_t$)[4].

The sign of the frequency of a coherent state $|X\rangle\langle Y|$ is defined, without loss of generality, to be positive if level $|X\rangle$ is higher in energy than level $|Y\rangle$, and negative if level $|X\rangle$ is lower. The exemplary Feynman diagrams reveal that, in the rephasing pathways, the coherent state evolution during τ is associated with a negative frequency, because the first interaction with a light field occurs from the right side, bringing the bra state from $\langle g|$ to $\langle e|$, while the coherent state during t evolves with positive frequency, because, in that case, the ket state has higher energy than the bra state. The different sign for the temporal evolution in the two coherence times is the reason why this is called the “rephasing” signal, since the phase evolution during the first period is reversed in the second. Therefore, in rephasing maps, $\hbar\omega_\tau$ assumes negative values[4]. By contrast, in nonrephasing pathways, the coherent states during both τ and t have positive frequencies.

We now consider the differences between the two types of 2DES from the Feynman diagrams in Supplementary Figs. 2,3. For the population-detected approach (Supplementary Fig. 2), there are four impulsive transitions, generating an excited population state (i.e., identical bra and ket states), followed by spontaneous emission of fluorescence. For the coherence-detected approach (Supplementary Fig. 3), three laser pulses are used to create a third-order polarization (i.e., different bra and ket states) which emits a coherent signal in the phase-

matched direction. Therefore, when measuring rephasing and nonrephasing 2d maps, population-detected 2DES probes fourth-order nonlinear signals, whereas in the coherence-detected geometry one records the third-order response of the system. The apparent discrepancy in the nonlinearity comes about because in the standard formulation of coherence-detected 2DES the light field is treated classically and the final interaction (dashed arrows) is not counted towards the order of nonlinear response[4]. Third-order coherently detected 2d spectra and fourth-order population-detected ones can both be described with one and the same underlying generalized fourth-order response function, discussed in Ref. 5.

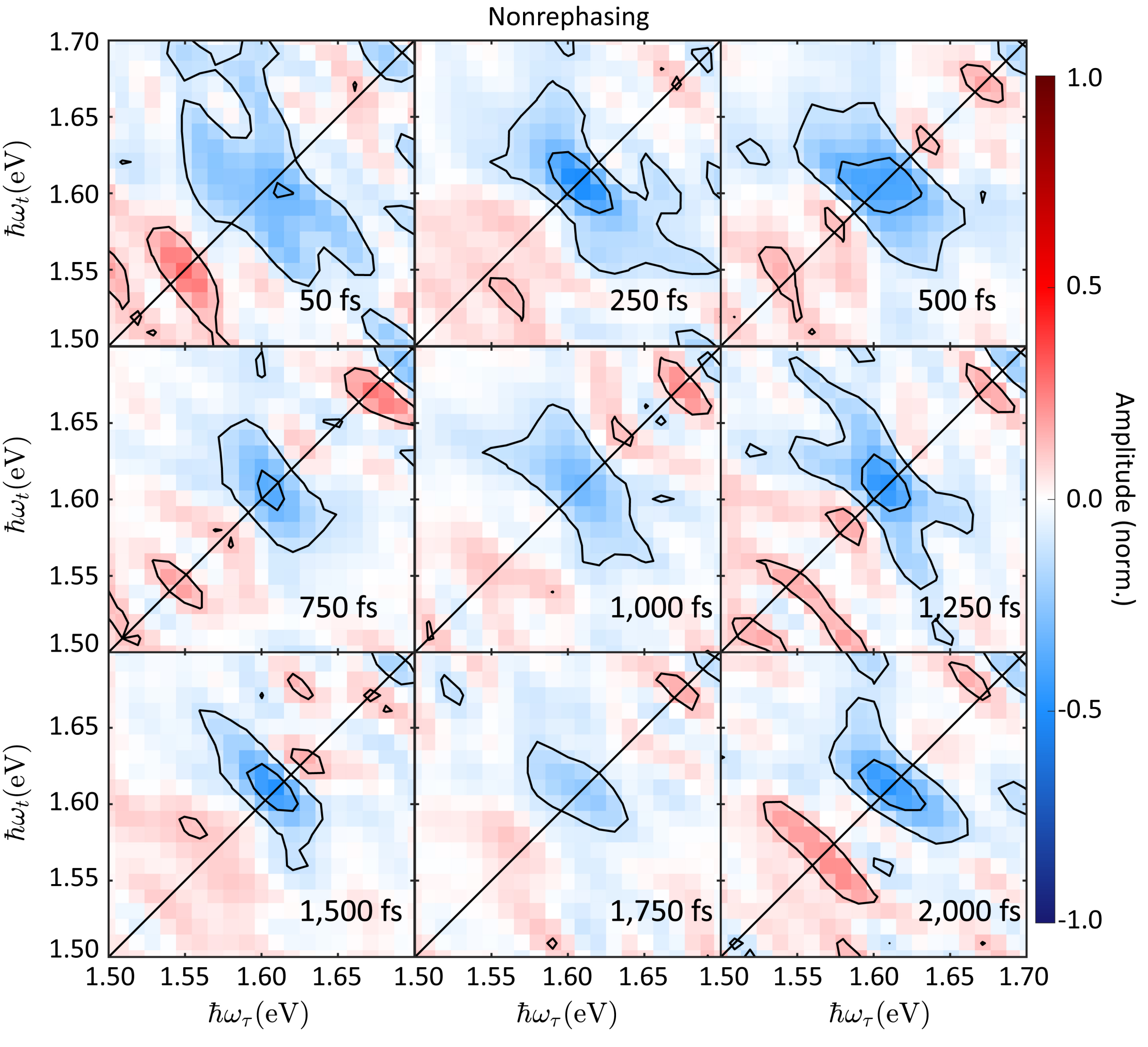

Supplementary Figure 1: Nonrephasing 2d maps (real parts) at different *T*.

In coherence-detected 2DES there are three types of Feynman pathways[4] contributing to rephasing and nonrephasing signals, named ground-state bleach (GSB), stimulated emission (SE), and excited-state absorption (ESA), as labeled in Supplementary Fig. 3, wherein the GSB and the SE pathways have positive sign, and the ESA negative. Conversely, in population-detected 2DES, the GSB and SE signals are negative (Supplementary Fig. 2), since there is always one more interaction from the right side compared to the coherence-detected variant. For the ESA signal of population-detected 2DES maps, two pathways exist (ESA 1 and ESA 2) ending up in |e⟩⟨e| and |f⟩⟨f| excited population states[6]. If the system arrives at the |f⟩⟨f|

state, fast internal conversion will lead to the $|e\rangle\langle e|$ state before spontaneous emission can occur (Kasha's rule[7]). Hence, ESA 1 and 2 have the same intensities, but opposite signs, under the condition of a unity quantum efficiency for the internal conversion process from $|f\rangle\langle f|$ to $|e\rangle\langle e|$ (i.e., if all the population of the $|f\rangle\langle f|$ state is transferred to the $|e\rangle\langle e|$ state). This results in a cancellation between the two ESA pathways in population-detected 2DES[6]. Such a cancellation is fulfilled here because, if ESA 2 would not fully cancel ESA 1, a left-over ESA signal would appear on the 2d maps at $\hbar\omega_\tau = \hbar\omega_{\mathrm{eg}}$ and $\hbar\omega_t = \hbar\omega_{\mathrm{fe}}$, where $\hbar\omega_{\mathrm{eg}}$ ($\hbar\omega_{\mathrm{fe}}$) is the transition energy between $|g\rangle$ and $|e\rangle$ ($|e\rangle$ and $|f\rangle$). Thus, henceforth all the ESA pathways are neglected. For molecular systems, the situation may be different, and one has to include a suitable less-than-unity quantum efficiency parameter and take into account ESA pathways[6,8,9].

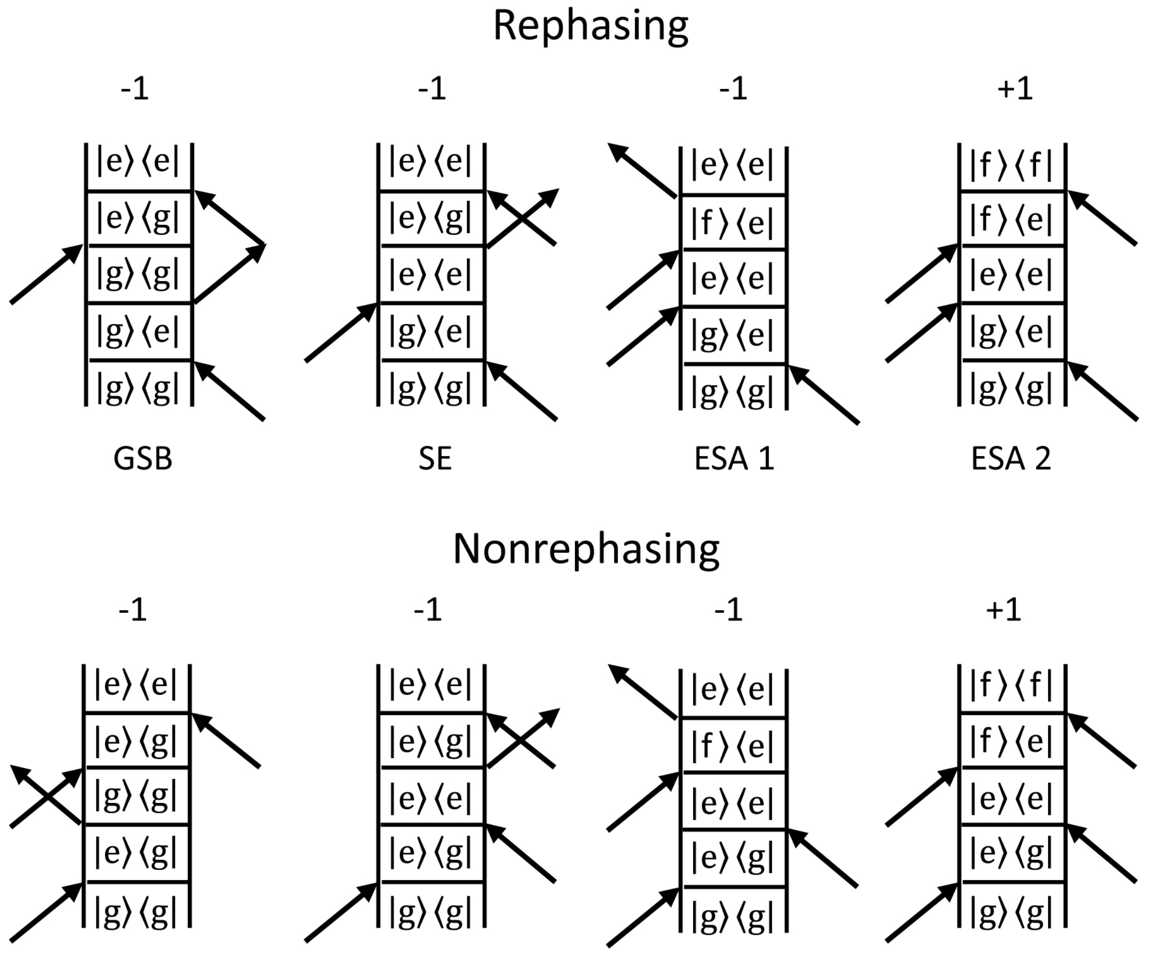

Supplementary Figure 2: Typical Feynman diagrams (without considering sublevels within electronic states) of rephasing (top row) and nonrephasing (bottom row) pathways in population-detected 2DES. There are four types of Feynman pathways contributing to rephasing and nonrephasing signals, named ground-state bleach (GSB), stimulated emission (SE), and excited-state absorption (ESA) 1 and 2.

Supplementary Note 2: Absorptive 2d maps and linewidth analysis

Upon Fourier transformation, both rephasing and nonrephasing signals have dispersive contributions[4]. In order to compare to static or transient absorption spectra, 2d rephasing and nonrephasing maps are normally summed up to cancel the dispersion and obtain an absorptive

lineshape[4], as illustrated in Supplementary Fig. 4. The resulting absorptive 2d maps at different T are in Supplementary Fig. 5, normalized to the maximum absolute value at $T = 500$ fs.

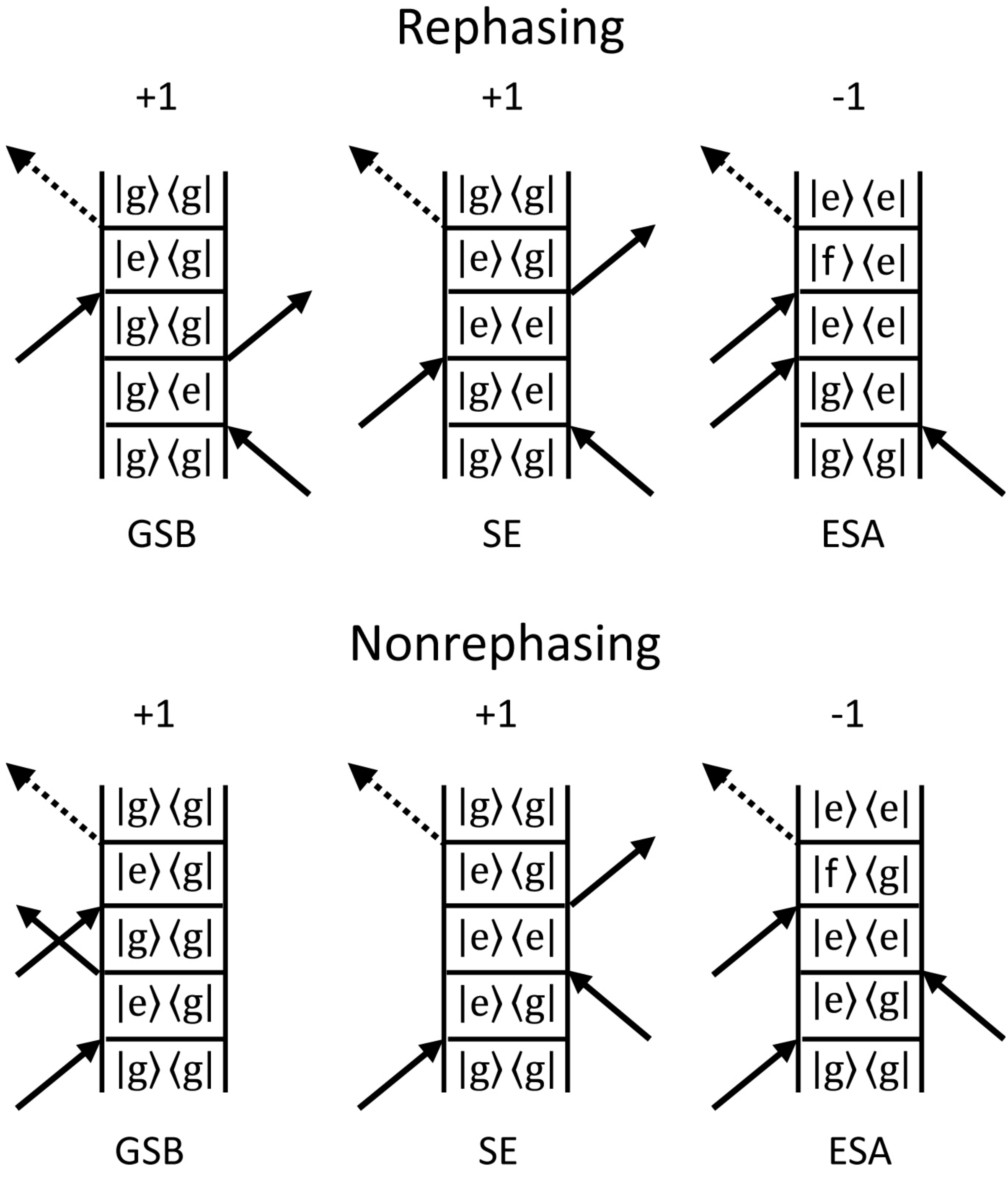

Supplementary Figure 3: Typical Feynman diagrams (without considering sublevels within electronic states) of rephasing (top row) and nonrephasing (bottom row) pathways in conventional coherence-detected 2DES. There are three types of Feynman pathways contributing to rephasing and nonrephasing signals, named ground-state bleach (GSB), stimulated emission (SE), and excited-state absorption (ESA).

Absorptive 2d maps simplify the interpretation and allow direct comparison with traditional transient absorption spectroscopy[4]. For studying the origin of coherent oscillations

as a function of T, it is preferable to analyze separately the amplitude evolutions in the rephasing and nonrephasing maps, because they display different oscillating behaviors for diagonal versus cross peaks. The summation of rephasing and nonrephasing signals will mix these different oscillations and obscure the analysis[10].

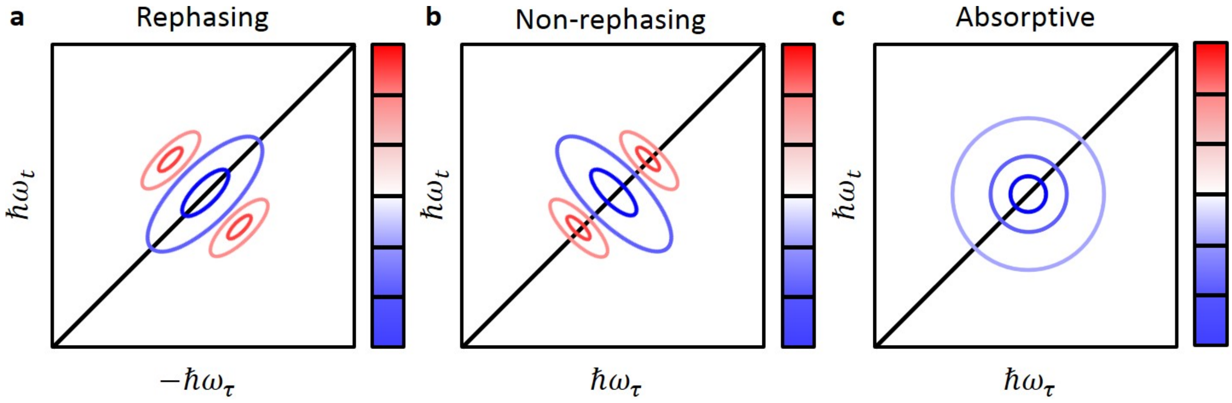

Supplementary Figure 4: Schematic real parts of **a**, rephasing, **b**, nonrephasing, and **c**, absorptive 2d maps in the case of a single relevant transition. Both rephasing and nonrephasing signals have dispersive contributions. The summation of their real parts can remove the dispersion, resulting in a purely absorptive map.

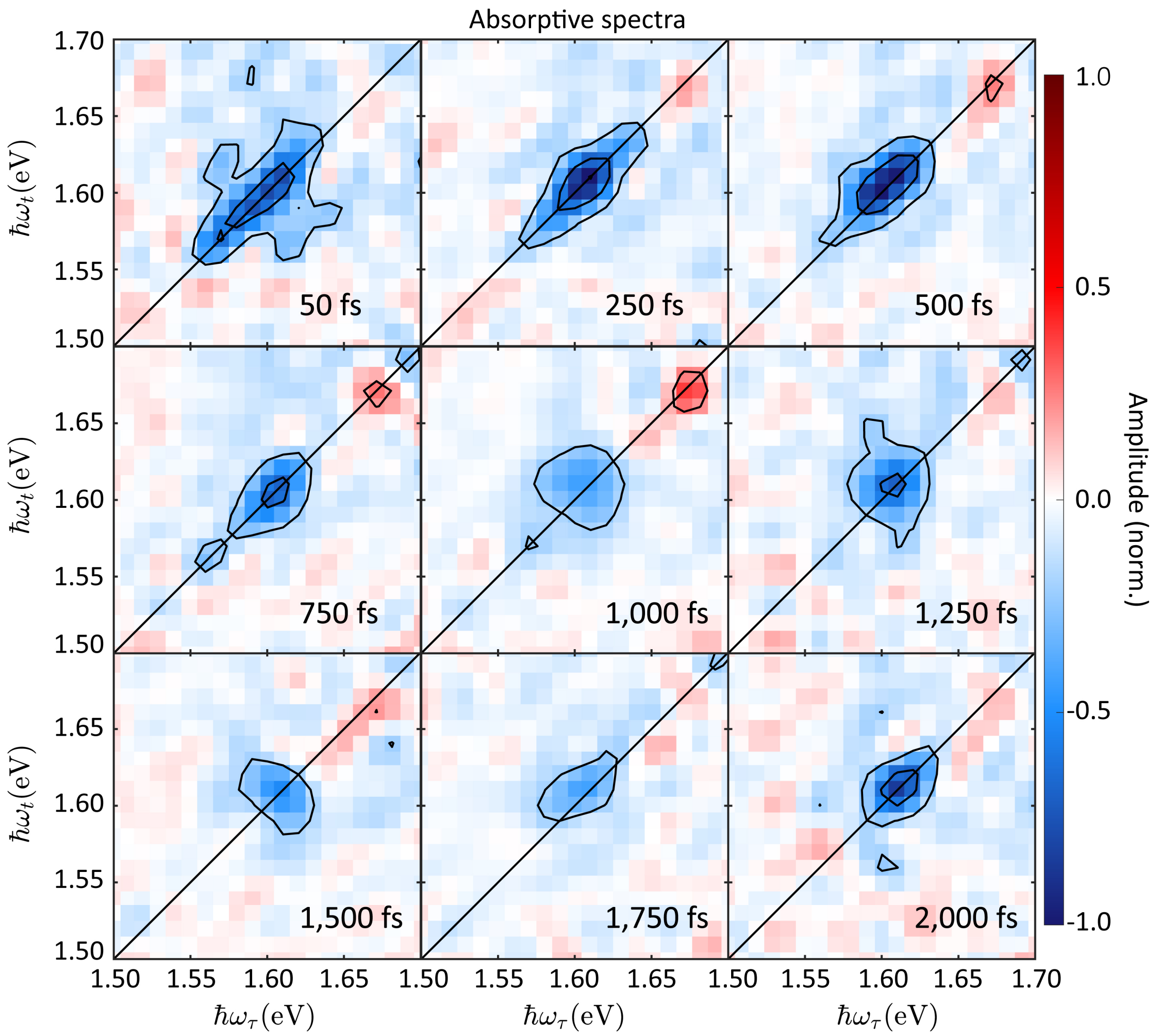

Supplementary Figure 5: Absorptive 2d maps at different T normalized to the maximum at $T = 500$ fs.

In the absorptive 2d maps (Supplementary Fig. 5), the linewidth of the peak along the diagonal direction oscillates with T (Supplementary Fig. 6a, black curve). The orange curve in Supplementary Fig. 6a is the diagonal linewidth of the rephasing maps (the same curve as in Fig. 2b in the main text), which shows the same trend as the black curve, indicating that there are multiple diagonal components. The amplitudes of these components oscillate, yet not in phase. Supplementary Figs. 6b (6c) show the absorptive map at T = 250 fs (1500 fs). By comparing the linewidth in diagonal direction (Supplementary Fig. 6d, solid red line for T = 250 fs and solid green line for T = 1500 fs), we find that at 1500 fs the amplitudes of components 1 and 3 drop to zero, leaving only component 2, due to the out-of-phase oscillation.

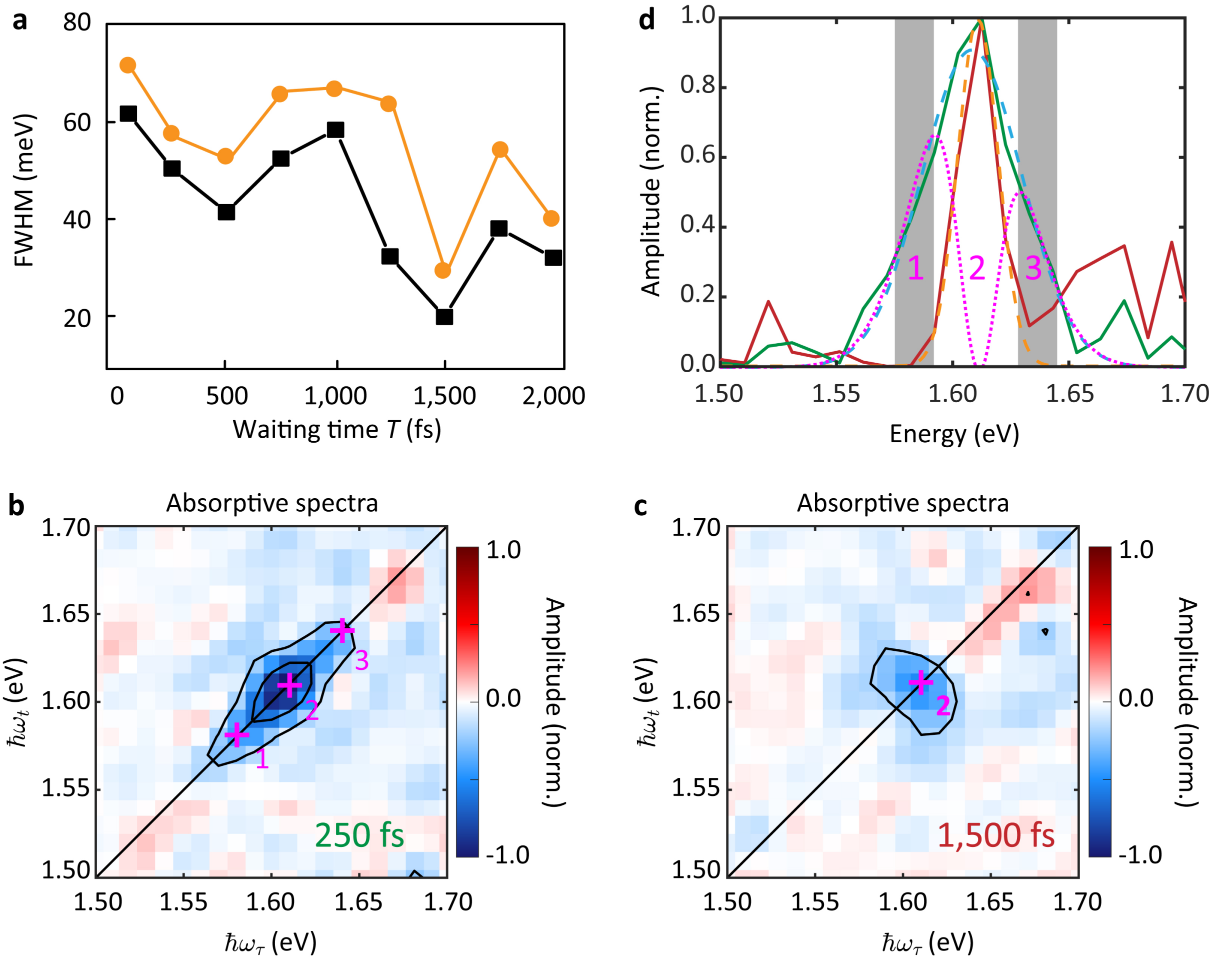

Supplementary Figure 6: a, FWHM along diagonal direction as a function of *T* from Gaussian fitting of each time step for the absorptive (black curve) and rephasing (orange curve) 2d maps. **b**, Absorptive 2d map at $T = 250$ fs. **c**, Absorptive 2d map at $T = 1500$ fs. **d**, Slices along diagonal direction for the 2d maps of panels b (solid green) and c (solid red), their Gaussian fitting curves (dashed orange and dashed green, respectively), as well as the difference of the two Gaussian curves (dotted purple). The gray areas mark the estimated ranges of the center positions of components 1 and 3 as labeled.

Both linewidths are fitted by Gaussians (Supplementary Fig. 6d, dashed orange line for $T = 250$ fs and dashed cyan-blue line for $T = 1500$ fs). The FWHM of component 2 is 20±8 meV, smaller than the FWHM ~50 meV derived from linear absorption at RT[11], because the peak intensity in 2d maps is proportional to the fourth power of the transition dipole strength[1], while linear absorption scales quadratically with transition dipole strength[4], resulting in a twice-smaller Gaussian linewidth in 2d maps than in linear ones. The laser spectrum additionally modulates the peak shape, and influences the comparison between the linewidth extracted from 2d maps and absorption spectra.

The position of component 2 is ~1.611±0.004 eV, as determined from the Gaussian fit at $T = 1{,}500$ fs, while the approximate positions of components 1 and 3 can be deduced from the difference of the two Gaussian fitting functions at T~ 250 and 1,500 fs (Supplementary Fig. 6d, dotted purple line). The two maximum positions in the difference spectrum are 1.591 and 1.629 eV. However, in the laser spectrum (Supplementary Fig. 7), there is a structured peak with maximum position~1.620 eV. Such a structure will shift the peak positions of different components towards the (local) laser peak maximum position (1.620 eV). Thus, the peak of

component 1 will be blue-shifted and component 3 red-shifted. For this reason, we cannot obtain precise center positions of components 1,3 just from maximum peak positions. We estimate their range by setting 1.591 eV (1.629 eV) as the upper (lower) limit of the position of component 1 (component 3), and the position at half the maximum on the low-energy (high-energy) side as the lower (higher) limit. The estimated ranges of the two components are marked by gray areas in Supplementary Fig. 6c. The energy splitting between components 1 and 2 falls in the range ~20–35 meV and, between 2 and 3, in the range ~18–33 meV. The estimated center positions of the diagonal peaks of components 1 and 3 are determined by taking the mid-point of each range, which are ($\hbar\omega_\tau$, $\hbar\omega_t$) = (1.584 eV, 1.584 eV) and (1.637 eV, 1.637 eV), respectively, and the estimated center position of the diagonal peak of component 2 [($\hbar\omega_\tau$, $\hbar\omega_t$) = (1.611 eV, 1.611 eV)], marked by purple crosses in Fig. 2a of the main text and in Supplementary Fig. 6.

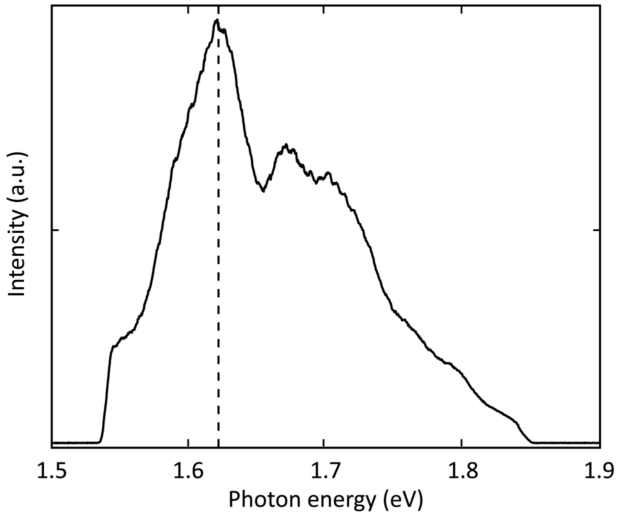

Supplementary Figure 7: Laser spectrum, with a structured peak with a maximum at 1.620 eV (dashed vertical line).

Supplementary Note 3: Exclusion of sixth-order signal

We perform a 27-step (1×3×3×3) phase-cycling scheme, whereby not only the time delays between the four laser pulses, but also the phases of individual pulses are scanned. The PL signal is proportional to the final excited-state population (where we may exclude ESA pathways as explained in Supplementary Note 1) resulting from all possible Feynman pathways, which can be described as[1]:

$$p(\varphi_1, \varphi_2, \varphi_3, \varphi_4) = \sum_{\alpha,\beta,\gamma,\delta} \tilde{p}_{(n)}\,(\alpha, \beta, \gamma, \delta)\exp[i(\alpha\varphi_1 + \beta\varphi_2 + \gamma\varphi_3 + \delta\varphi_4)], \quad (1)$$

where φ_1, φ_2, φ_3, φ_4, are the phases associated with the four excitation pulses and $\tilde{p}_{(n)}$ denotes the nth-order contribution. The summation is carried out over integers α, β, γ, δ within a range of $-\infty$ to $+\infty$ for each parameter, subject to the condition:

$$\alpha + \beta + \gamma + \delta = 0. \quad (2)$$

A parameter combination [α, β, γ, δ] defines one specific nonlinear signal. E.g., [-1, 1, 1, -1] defines the fourth-order rephasing and [1, -1, 1, -1] the nonrephasing signal. To extract the signal with a specific contribution of [α, β, γ, δ] we take a discrete Fourier transform[1]:

$$\tilde{p}_{(n)}(\beta,\gamma,\delta\,) = \frac{1}{LMN}\sum_{n=0}^{N-1}\sum_{m=0}^{M-1}\sum_{l=0}^{L-1} p(l\cdot\Delta\varphi_{21}, m\cdot\Delta\varphi_{31}, n\cdot\Delta\varphi_{41})$$

$$\times\exp[-i(l\beta\cdot\Delta\varphi_{21} + m\gamma\cdot\Delta\varphi_{31} + n\delta\cdot\Delta\varphi_{41})], \tag{3}$$

where $L = 3$, $M = 3$, $N = 3$, are the numbers of steps we scan within a 2π range for the phase of each pulse, and $\Delta\varphi_{21}$, $\Delta\varphi_{31}$, $\Delta\varphi_{41}$ are the increments of the phase steps. In Supplementary Equation 3, α is missing because in the 1×3×3×3 phase-cycling scheme we fix $\varphi_1 = 0$, since the signal only depends on relative phase, i.e., $\varphi_{i1} = \varphi_i - \varphi_1$, for $i = 2, 3, 4$.

In principle, 27-step phase cycling cannot exclude sixth-order signals. When extracting the desired fourth-order rephasing signal, we also obtain four types of sixth-order signals at the same time. They arise from combinations of [α, β, γ, δ] as[1]:

$$\alpha = +2, \beta = -2,\ \gamma = +1, \delta = -1, \tag{4}$$

$$\alpha = -1, \beta = -2,\ \gamma = +1, \delta = +2, \tag{5}$$

$$\alpha = +2, \beta = +1,\ \gamma = -2, \delta = -1, \tag{6}$$

$$\alpha = -1, \beta = +1,\ \gamma = -2, \delta = +2. \tag{7}$$

Similarly, when extracting the desired fourth-order nonrephasing signal, we also obtain four types of sixth-order signals at the same time. Their [α, β, γ, δ] combinations are[1]:

$$\alpha = -2, \beta = +2,\ \gamma = +1, \delta = -1, \tag{8}$$

$$\alpha = +1, \beta = +2,\ \gamma = -2, \delta = -1, \tag{9}$$

$$\alpha = -2, \beta = -1,\ \gamma = +1, \delta = +2, \tag{10}$$

$$\alpha = +1, \beta = -1,\ \gamma = -2, \delta = +2. \tag{11}$$

Although sixth-order signals normally are much weaker than fourth-order ones, as predicted by perturbative response function theory[12], they might still influence our analysis of the oscillating behavior of 2d maps. In order to examine any influence of the sixth-order signal overlapping with the fourth-order rephasing and nonrephasing signals, we conduct a separate measurement employing 64-step (1×4×4×4) phase cycling for T = 50 fs. This allows us to separate the fourth-order rephasing and nonrephasing maps from eight sixth-order signals. The resulting absolute-valued 2d maps corresponding to these ten contributions are in Supplementary Fig. 8. The rephasing and nonrephasing fourth-order maps (left column) are consistent with those measured using 27-step phase cycling. There is no detectable sixth-order signal above the noise floor (right four columns) that would overlap with the fourth-order signals. Therefore, the oscillations as a function of T, discussed in the main text, arise from rephasing and nonrephasing fourth-order pathways without any higher-order signal.

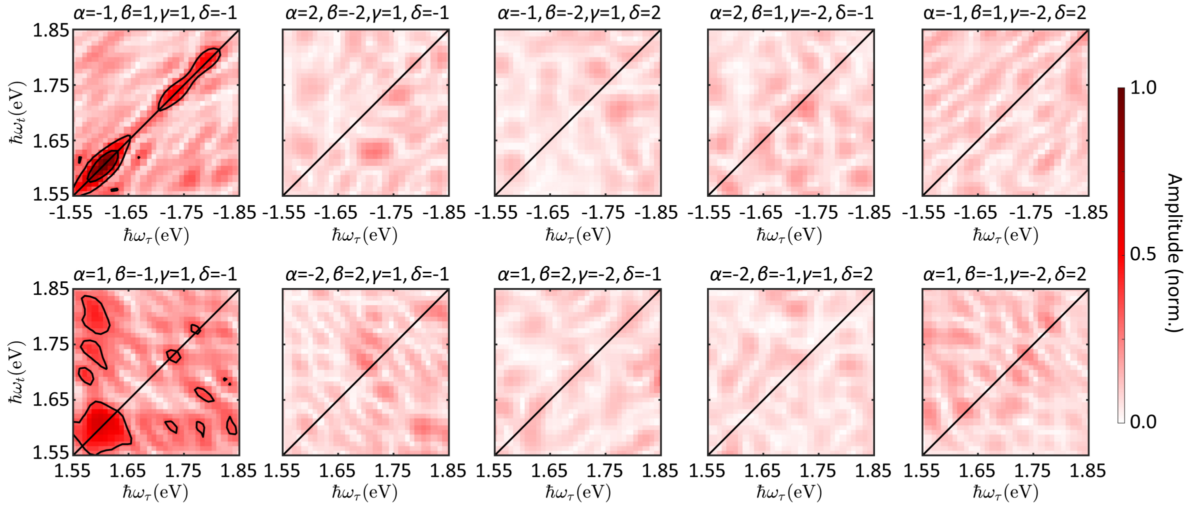

Supplementary Figure 8: Absolute-valued 2d maps of ten nonlinear contributions at $T = 50$ fs with 64-step phase cycling. [α, β, γ, δ] are labeled on top of the corresponding maps. The top left graph corresponds to the fourth-order rephasing signal and the bottom left one to the fourth-order nonrephasing one. There is no detectable signal beyond the noise floor for any of the other eight sixth-order nonlinear contributions.

Supplementary Note 4: Reproducibility and noise-level analysis

The reproducibility of the 2d maps is confirmed by twice repeated 2DES measurements. The top two rows of Supplementary Fig. 9 show the rephasing 2d maps for $T = 50$, 500, 1000 fs measured on two different experimental runs (labeled A for the first and B for the second row) for ~20 μJ/cm^2. The 6 maps are all normalized to the maximum absolute value of the real part of the upper rephasing map for $T = 50$ fs. The evolution of the peak shape with T is consistent for the two measurements. The difference maps of A and B (third row of Supplementary Fig. 9) show only background noise, indicating that the contributing signals reproduce each other, thus cancelling each other in the difference.

The reproducibility can be better illustrated by comparing the amplitude evolutions of a representative pixel (marked by the green diamond in the upper left panel of Fig. 2a of the main text) as a function of T for the two measurements (Supplementary Fig. 10). The green curve (the first run) and the blue curve (the second run) agree with each other.

The difference map between two measurements for the same T provides a way to evaluate the noise level, by evaluating the standard deviation (SD) of the data. However, this inherits the noise from both maps, hence we need to estimate correctly the signal-to-noise ratio of each individual map.

Supplementary Figs. 11a,b are zoomed-out rephasing maps for $T = 50$ fs, corresponding to the data sets in the first column of Supplementary Fig. 9. We analyze separately the noise level of each map by evaluating SD outside the signal region marked by the dashed orange box, yielding 0.0745 and 0.0788 for panels a and b, respectively.

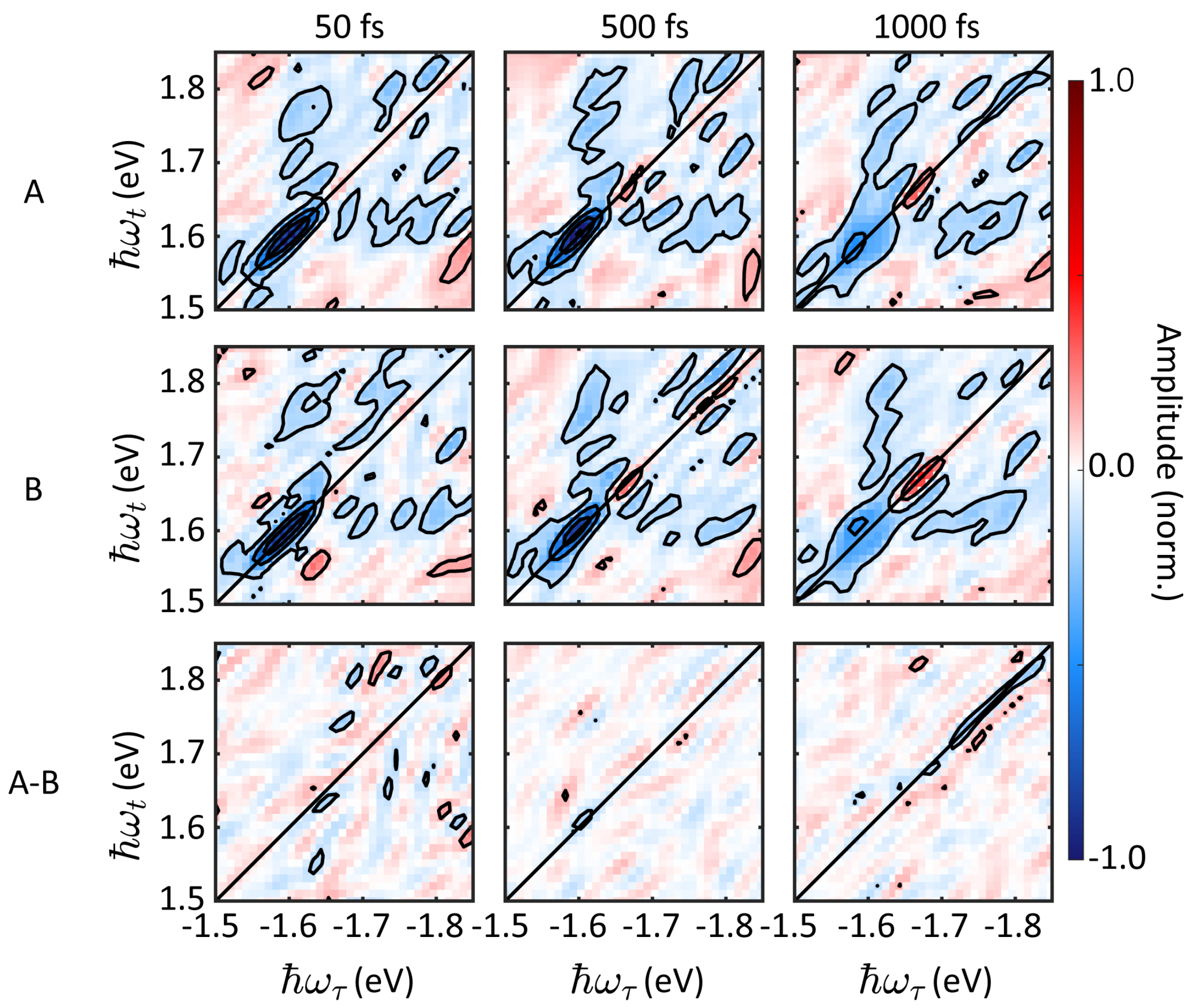

Supplementary Figure 9: Reproducibility of rephasing 2d maps at T = 50, 500, 1000 fs. The row labeled A contains a first and row B a second set of measurements. The evolution of peak shape with respect to T is consistent for the two measurements. Row A-B contains the difference between A and B. The signals largely cancel, leaving only background noise.

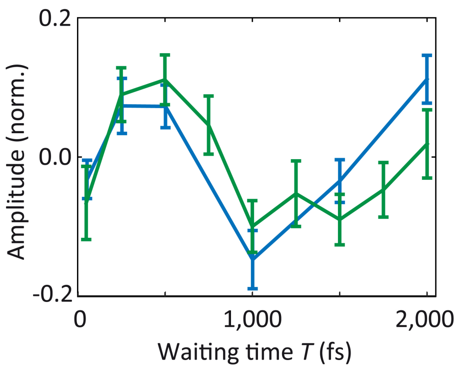

Supplementary Figure 10: Reproducibility of time traces for the same position within the rephasing 2d maps measured in two repeated runs. The error bars are evaluated by calculating the fluctuations within a region containing background noise.

The difference map is in Supplementary Fig. 11c. SD inside the dashed orange box of the difference map is 0.0686, close to the calculated SD from panels a, b outside of the orange box. The agreement between SD from the outside-box region in panels a, b and from the inside-

box region in panel c indicates that the noise is evenly distributed. Thus, the SD calculated outside the signal region can be used to evaluate the fluctuation ranges of the measured amplitude for each single pixel in the signal region. For each T, we separately extract SD, and use it to create an error bar for the corresponding T step in the amplitude evolution curves in Supplementary Figs. 10,12 and Fig. 2c of the main text.

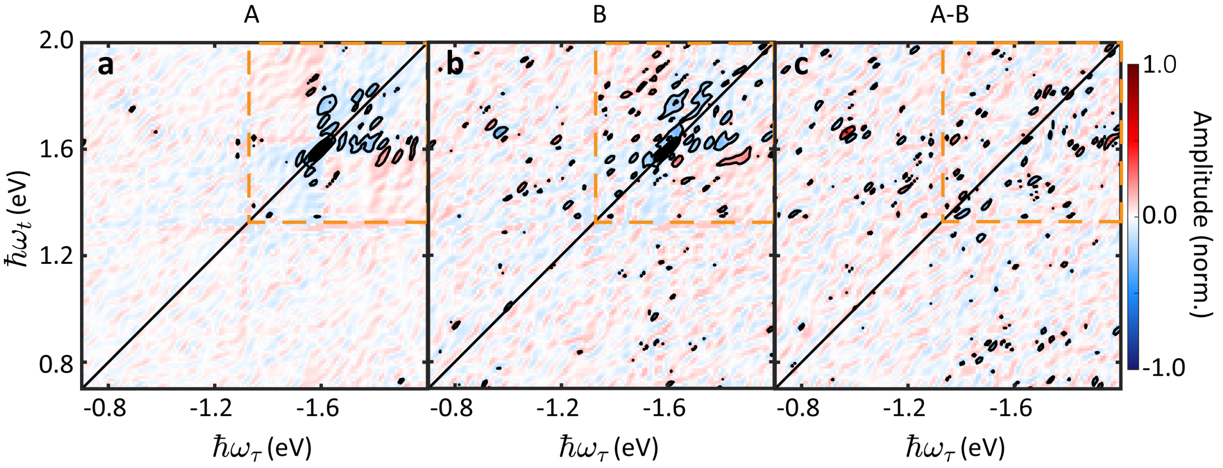

Supplementary Figure 11: Noise-level analysis. **a**,**b**, Zoomed-out rephasing 2d maps at T = 50 fs of the first column of Supplementary Fig. 9 for (a) row A and (b) row B. The dashed orange boxes mark the signal regions in the two maps. **c**, Difference between panels a and b.

Supplementary Note 5: Exclusion of biexciton signal

Biexcitons have a binding energy ~20 meV[13–15]. In view of the Feynman-pathway analysis of Supplementary Figure 2, biexcitons can only be detected through excited-state absorption (ESA) pathways in a 2d spectroscopy measurement, meaning that they appear in 2d maps as a peak outside the diagonal and at lower probe energies (~20 meV) compared to the neutral exciton[13]. This should correspond to a strongly asymmetric lineshape towards the red (low detection frequency ω_t) in the absorptive 2d maps. We do not observe this, indicating that the effect of biexcitons is negligible in our experiments at room temperature.

The following two factors could explain this: 1) The binding energy of the neutral biexciton is an order of magnitude lower than the exciton binding energy[16,17]. Thermal fluctuations make biexcitons unstable, and lead to biexciton dissociation at RT. Therefore, upon laser excitation, even if some biexcitons are present at RT, we expect their spectral signal to be much weaker than the single neutral exciton. 2) As discussed in Supplementary Note 1, in population-detected 2d spectroscopy, the two types of excited-state absorption (ESA) pathways, i.e., ESA 1 and ESA 2, usually cancel each other to some extent (depending on their associated fluorescence quantum yields), leading to a reduction of ESA signal, hence a further reduction of the contribution from biexcitons.

Supplementary Note 6: Extracting the oscillation period

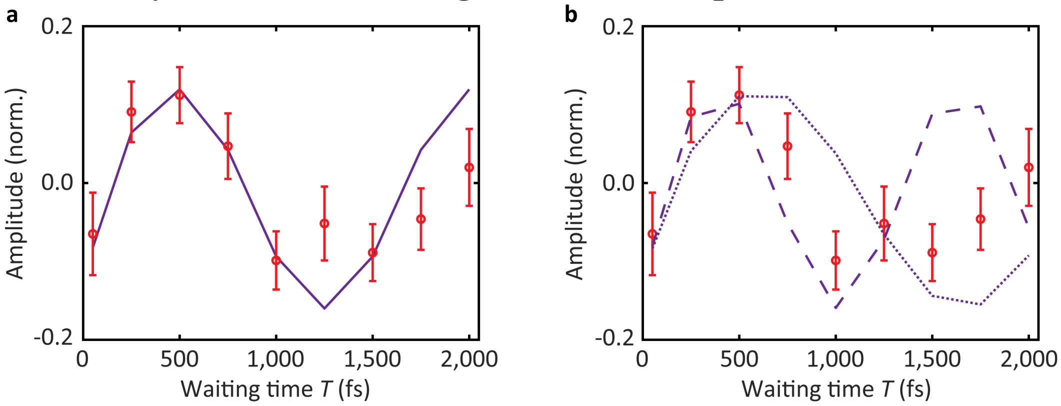

Supplementary Figure 12: a, Fitted oscillating curve (solid purple). **b**, Representative sinusoidal curves with varied oscillation periods of 133 fs (dotted) and 139 fs (dashed) yielding significant deviations with respect to the measurements, thus demonstrating the accuracy of the fitting of panel a. The error bars are evaluated by calculating the fluctuations within a region containing background noise (Supplementary Note 4).

To extract the oscillation period from the amplitude evolution curve (shown in Fig. 2c in the main text), we fit it using a sine function:

$$Y = A \sin\left[\frac{2\pi(x - x_0)}{w}\right], \tag{12}$$

with amplitude A, phase x_0, and period w restricted to ~118–230 fs (as obtained from the positions of the constituent components). An oscillation period w~136 fs is obtained from the fitting. Supplementary Fig. 12a shows the fitting results, where the measured data are plotted as red circles with error bars. The purple curve is obtained by taking only those time points (50, 250, 500, 750, 1000, 1250, 1500, 1750, 2000 fs) at which 2d maps are measured, and connecting the values obtained from the fitting function by straight line segments. The accuracy of the extracted period is checked by varying it and comparing the resulting curves with the data. As shown in Supplementary Fig. 12b, if we change the period to either 133 fs (dotted purple line) or 139 fs (dashed purple line), the curves deviate strongly from the data, from which we derive the ±2 fs error of the main text.

Supplementary Note 7: Definition of Huang–Rhys factor *S*

We consider an electronic (or excitonic) ground state $|g\rangle$ and an electronic (or excitonic) first excited state $|e\rangle$. Using a harmonic oscillator to approximate the dependence of potential energy on a vibrational (phonon) dimensionless coordinate q with the ground-state minimum at $q = 0$ [12],

$$V_g(q) = \frac{\hbar\omega}{2} q^2, \tag{13}$$

the potential curvature leads to a vibrational level spacing of $\hbar\omega$, where ω is the phonon angular frequency, creating sublevels $|g_i\rangle$, i = 0, 1, 2, …. For full information on the system, we also need to describe the excited-state potential[12],

$$V_e(q) = \hbar\omega_{eg} + \frac{\hbar\omega}{2}(q+d)^2, \tag{14}$$

in which we assume, for simplicity, the same curvature, thus the same $\hbar\omega$ as in the ground state, a (vertical) energy difference $\hbar\omega_{\mathrm{eg}}$, and a (horizontal) shift along the phonon coordinate that can be formulated in a dimensionless way, *d*, between the two potential minima. This generates sublevels $|\mathrm{e_i}\rangle$, i = 0, 1, 2, …. Substituting Supplementary Equation 13 into Supplementary Equation 14 leads to

$$V_e = \hbar\omega_{eg} + V_g + \hbar\omega S + \hbar\omega q d, \tag{15}$$

where $S = d^2/2$ is the Huang–Rhys factor, and $\hbar\omega S$ is the reorganization energy.

Supplementary Note 8: Peak position of individual Feynman pathways

The peak position along the (horizontally displayed) $\hbar\omega_\tau$ excitation energy axis can be found by evaluating the energy difference between the states of the coherence created after the first light-field interaction. E.g., considering pathway 1, the first coherence created (and evolving with τ) is $|\mathrm{g_0}\rangle\langle\mathrm{e_0}|$. According to Fig. 3a of the main text (black arrow), this is located at the intermediate of the three possible transition energies for the excitation, i.e., at $\hbar\omega_\tau$, where the minus sign in Fig. 3e of the main text arises from the definition of sign of the frequency of a coherent state (a coherent state $|\mathrm{X}\rangle\langle\mathrm{Y}|$ has positive frequency when level $|\mathrm{X}\rangle$ is higher in energy than level $|\mathrm{Y}\rangle$ and negative frequency if level $|\mathrm{X}\rangle$ is lower than $|\mathrm{Y}\rangle$, see Supplementary Note 1). Likewise, the coherence after the third interaction (and evolving with *t*) is $|\mathrm{e_0}\rangle\langle\mathrm{g_1}|$, at the lowest of the 3 transition energies $\hbar\omega_1$ for the detection, according to the blue arrow in Fig. 3a of the main text with positive sign because $|\mathrm{e_0}\rangle$ is higher than $|\mathrm{g_1}\rangle$. All other peaks are assigned in a similar way, so that the displayed pattern emerges.

Supplementary Note 9: 2d beating maps for different *S* values

Supplementary Figure 13 contains simulated 2d beating maps for various *S*, a subset of which is shown in Fig. 4a of the main text. The lowest contour lines of the experimental and the simulated beating maps in Figs. 4a,b of the main text and Supplementary Figure 13 show some "jagged" behaviour. There are several factors that could contribute to this. 1) The measurement uncertainty arising from noise becomes more visible at the lowest contour line for any given signal-to-noise level, leading to deviations from an ideal elliptical shape. 2) The energy resolution is given by the temporal scanning range and is ~40.6 meV. Using additional four-fold zero padding, one pixel has a side length ~10 meV, corresponding to 20 frequency pixels in the spectral ranges displayed in Fig. 4 of the main text, along either frequency axis. Thus, any (random) deviation, due to noise, in just one or two neighbouring independent frequency intervals will lead to a "jagged" outline of the respective contour line, because there are only few points that make up any such line. 3) The beating maps represent cuts through a three-dimensional Fourier space for a particular ω_T. However, the experimental scanning procedure sets a finite resolution along the ω_T direction, and any beating contribution has a finite width along this axis. Thus, it is possible that contributions from several different beating frequencies overlap at any given ω_T. If different beating contributions are located at different (ω_τ, ω_t), their interference can lead to a more complex appearance of the beating map for any particular ω_T

cut position. 4) The spectra are influenced by the shape of the excitation laser spectrum. If this spectrum deviates from a perfectly smooth function (such as a Gaussian), this will introduce additional structure. For optimal comparison between theory and experiment, we use the experimental spectrum also for simulations, thus jagged contour lines can emerge even in simulations without noise.

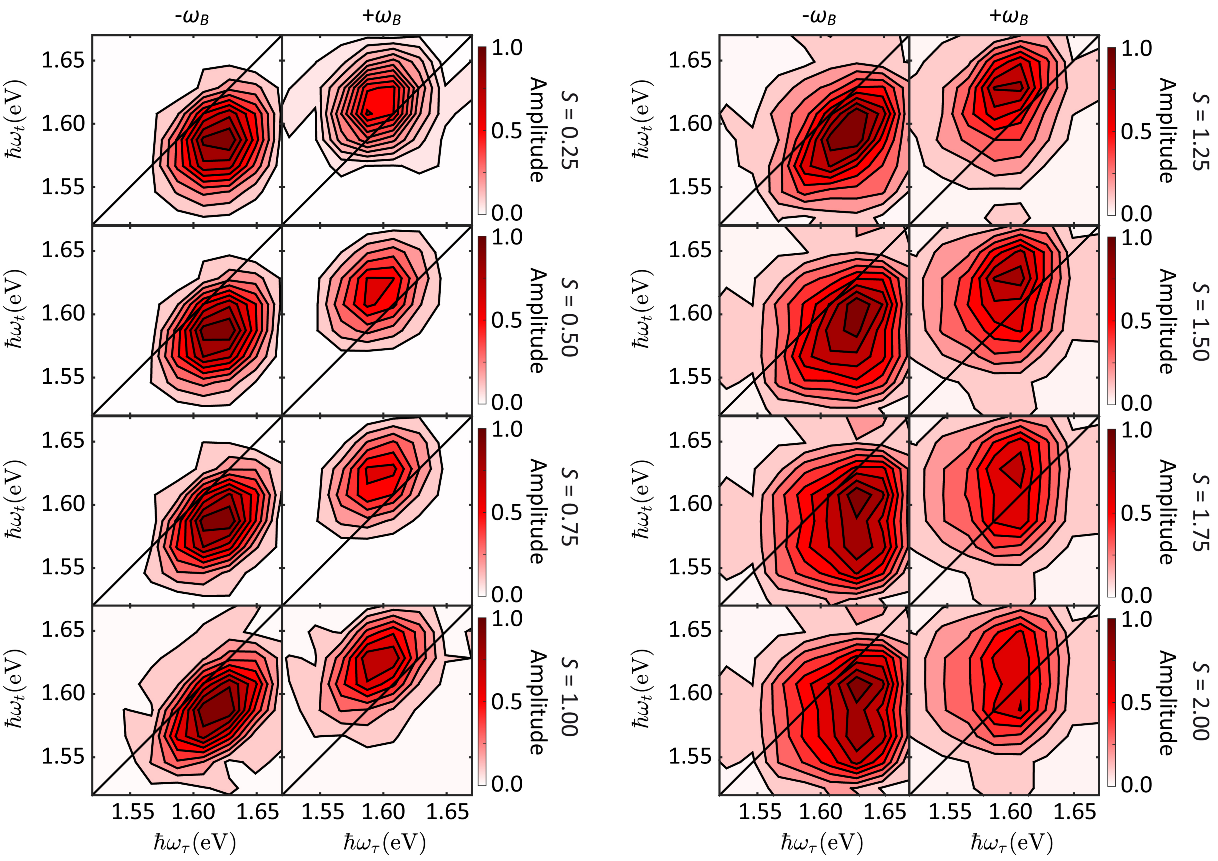

Supplementary Figure 13: Simulated 2d beating maps for $-\omega_B$ (left side in each double column) and $+\omega_B$ (right side in each double column) and for different S values.

Supplementary Note 10: Sample temperature during 2DES measurement

Heating the sample through laser irradiation during the experiment may cause thermal instabilities or damage. In addition, sample temperature is a decisive factor for calculating the 2d beating maps, because it determines the distribution of the initial population of the ground and excited vibrational states. We estimate the sample temperature during the 2DES measurements by adapting the two-temperature model[18], for the coupling of electronic and vibrational degrees of freedom in solids. This describes the energy transfer inside a material with two coupled generalized heat conduction equations for the temperature of the electrons T_e and the lattice T_l,

$$c_e \frac{\partial T_e(r,t)}{\partial t} = k_e \frac{\partial^2 T_e(r,t)}{\partial r^2} - \alpha[T_e(r,t) - T_l(r,t)] + \sigma \cdot I(r,t), \tag{16}$$

$$c_l \frac{\partial T_l(r,t)}{\partial t} = k_l \frac{\partial^2 T_l(r,t)}{\partial r^2} + \alpha[T_e(r,t) - T_l(r,t)] - \beta[T_l(r,t) - T_s], \tag{17}$$

where r is a spatial lateral coordinate, t the time, c_e and c_l are the electron and lattice volumetric heat capacities, k_e and k_l are the electron and lattice thermal conductivities, $\alpha = c_e/\tau_r$ is the thermal coupling function between electron and lattice subsystems, and τ_r the characteristic time of electron gas cooling due to energy exchange with the lattice[18]. β characterizes the rate of energy exchange between 1L-$MoSe_2$ and substrate. This can be expressed as the product of the interfacial thermal conductance h_c between 1L-$MoSe_2$ and substrate and the laser-irradiated area A. $I(r,t)$ is the intensity of the laser beam, σ is the absorbance of the sample. We set $T_s = 300$ K, by assuming infinitely fast heat dissipation from the substrate to surrounding areas.

To simplify the calculation, we ignore transverse thermal diffusion by setting $k_e = k_l = 0$, so that the absorbed laser energy is confined in the irradiated volume $V = A \cdot l$ before it is transferred to the substrate (l is the thickness of 1L-$MoSe_2$), and Supplementary Equations (16) and (17) reduce to

$$c_e \cdot V \cdot \frac{\partial T_e(t)}{\partial t} = -\left(\frac{c_e}{\tau_r}\right) \cdot V \cdot [T_e(t) - T_i(t)] + \sigma \cdot I(t), \tag{18}$$

$$c_i \cdot V \cdot \frac{\partial T_i(t)}{\partial t} = \left(\frac{c_e}{\tau_r}\right) \cdot V \cdot [T_e(t) - T_i(t)] - h_c \cdot A \cdot [T_i(t) - T_s]. \tag{19}$$

To take into account the cumulative effects owing to the high repetition frequency (80 MHz) of the laser, we consider a multi-pulse heating model[19]:

$$I(t) = \sum_{n=0}^{+\infty} I_0 \cdot e^{-\frac{(t-n\cdot t_i)^2}{w^2}} \tag{20}$$

for the laser intensity irradiating the material, with I_0 representing the peak intensity, w the duration of every laser pulse. The time interval t_i = 12.5 ns between individual pulses is defined by the repetition frequency. The integer number n ranges from zero to infinity, so that the sample is continuously heated from pulse to pulse. Because the laser power of the 2DES measurement is much lower than the damage threshold of the material, we calculate c_e using[18]:

$$c_e = \gamma \cdot T_e, \tag{21}$$

where γ is a proportionality constant that connects the heat capacity of the electron gas with its temperature. The values for all parameters in Supplementary Eq. 18–21 are in Supplementary Table 1.

Supplementary Fig. 14a plots the evolution of T_e (red curve) and T_l (blue curve) within one interval (12.5 ns) between two laser pulses. Upon arrival of the first pulse, T_e starts to increase since electrons are excited. Then the energy is transferred from electrons to lattice, resulting in a subsequent rise of T_l. The lattice finally gives its energy to the substrate because of the thermal contact between them, hence T_l decreases. If the interfacial thermal conductance h_c is high enough, T_l will drop back to the initial temperature before the next pulse comes, thus the same circle will repeat between any two pulses. On the other hand, if the heat released to the lattice does not have time to fully dissipate to the substrate before the next pulse arrives, the cumulative effects will lead to an increase of T_l from pulse to pulse until an equilibrium value is reached, as shown in Supplementary Fig. 14b, that plots T_l at the arrival time of pulse number n ($n = 1, 2, 3, ...$). Calculations indicate that, for ~3.6×10^{-14} J used in our 2DES measurements, T_l increases from 300 to ~308 K within the first 100 ns, then remains constant. Thus, there is no unwanted heating of the sample, thermal instabilities or damage.

There are two key assumptions in our calculation. 1) We assume that the absorbed energy is confined within the region of the laser focus, and does not diffuse to surrounding areas. 2) From a 1–2 orders of magnitude disagreement in the literature on h_c of TMDs (ranging between 0.1 to 14 MW m^{-2} K^{-1} [20–22]), we use the minimum in our calculation, so that the maximum T_l can be calculated. Because both assumptions overestimate the equilibrium T_l, these ensure that the experimental T_l does not exceed the calculated ~308 K, with a negligible heating during our measurements.

Supplementary Table 1: Parameters used in the TTM calculations.

	Value		Value
c_l	1.87×10^6 Jm^{-3}K^{-1} [23]	σ	2.5% [24]
A	5.3×10^{-14} m^2	I_0	2.8 W
l	0.65 nm	T_r	12.5 ns
τ_r	240×10^{-15} s [18]	t_c	100 fs
h_c	0.1 MWm^{-2}K^{-1} [21]	w	7.2 fs
γ	67.6 J m^{-3} K^{-2} [18]		

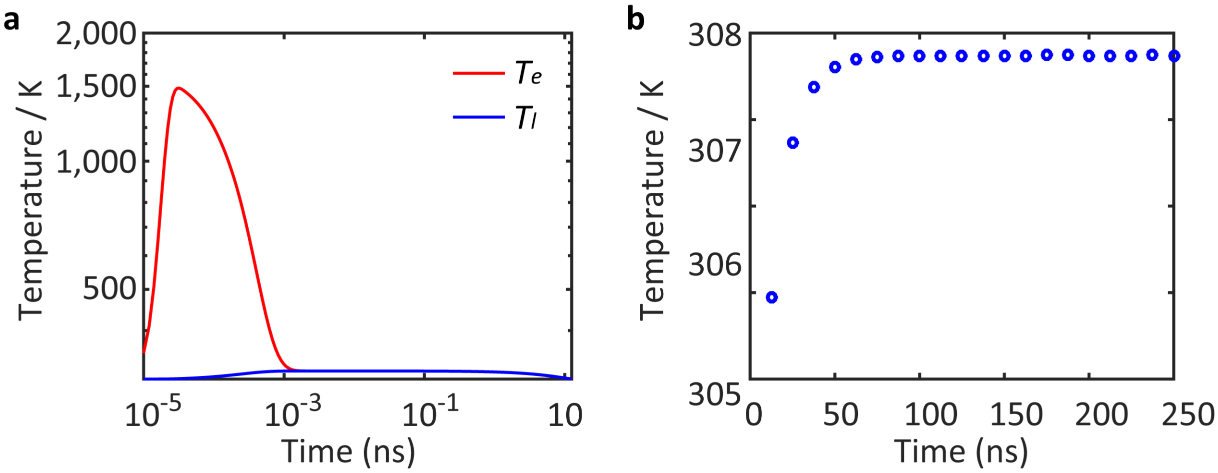

Supplementary Figure 14: a, Evolution of the temperature of the electrons T_e (red curve) and the temperature of the lattice T_l (blue curve) within one interval (12.5 ns) between two laser pulses. **b,** Calculated T_l at the arrival time of pulse n (n = 1, 2, 3, …).

Supplementary Note 11: Autocorrelation measured at the sample position

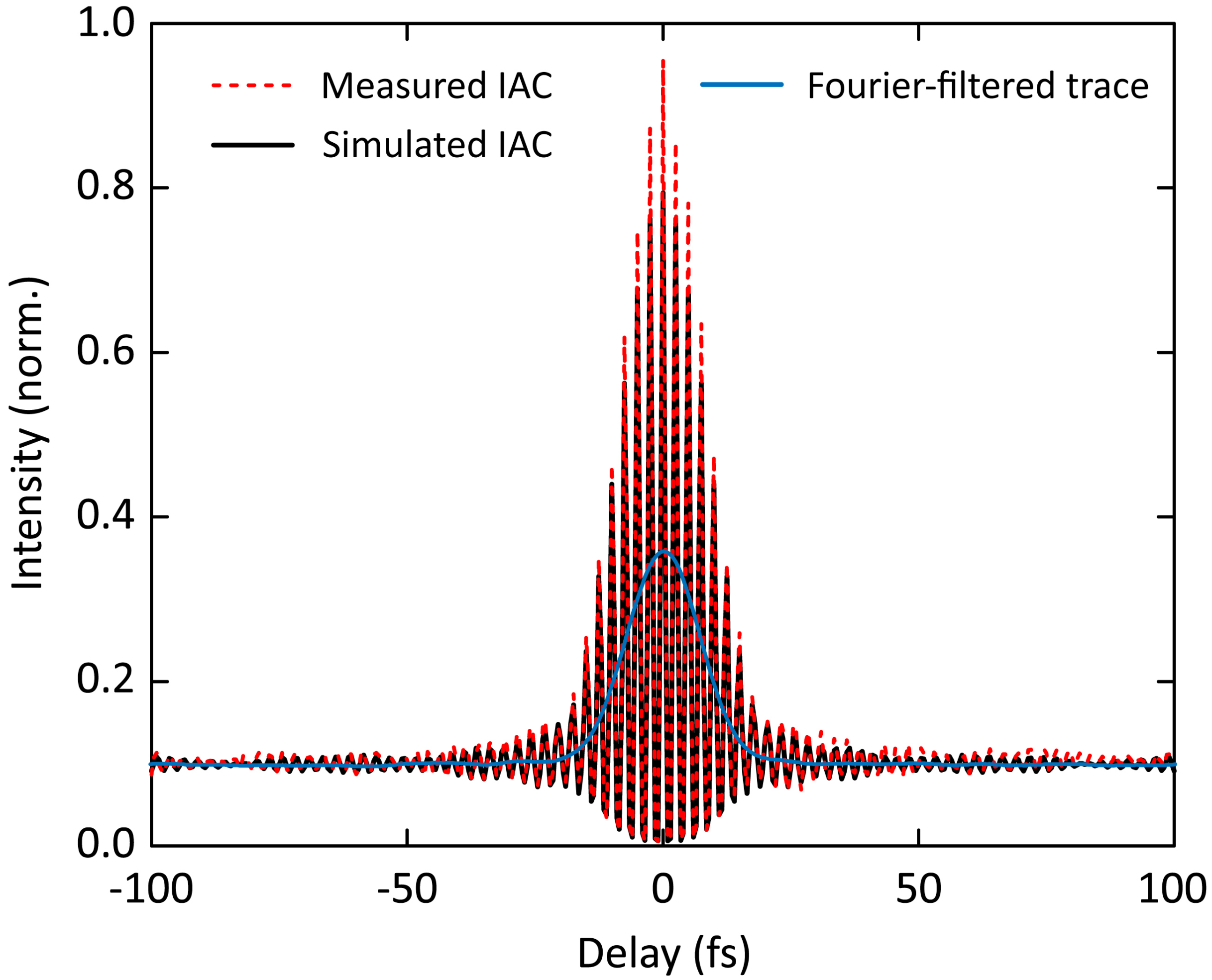

Supplementary Figure 15: Measured (dashed red) and simulated (solid black) interferometric autocorrelation (IAC) assuming a flat spectral phase and the separately measured laser spectrum. The pulse duration of ~12 fs can be obtained by dividing the FWHM of the Fourier-filtered trace by $\sqrt{2}$ (solid blue curve)[3].

Supplementary References

1. Tan, H.-S. Theory and phase-cycling scheme selection principles of collinear phase coherent multi-dimensional optical spectroscopy. *J. Chem. Phys.* **129**, 124501 (2008).
2. Draeger, S., Roeding, S. & Brixner, T. Rapid-scan coherent 2D fluorescence spectroscopy. *Opt. Express* **25**, 3259–3267 (2017).
3. Goetz, S., Li, D., Kolb, V., Pflaum, J. & Brixner, T. Coherent two-dimensional fluorescence micro-spectroscopy. *Opt. Express* **26**, 3915–3925 (2018).

4. Hamm, P. & Zanni, M. *Concepts and methods of 2D infrared spectroscopy*. (Cambridge University Press, 2011).
5. Malý, P., Mueller, S., Lüttig, J., Lambert, C. & Brixner, T. Signatures of exciton dynamics and interaction in coherently and fluorescence-detected four- and six-wave-mixing two-dimensional electronic spectroscopy. *J. Chem. Phys.* **153**, 144204 (2020).
6. Malý, P. & Mančal, T. Signatures of exciton delocalization and exciton–exciton annihilation in fluorescence-detected two-dimensional coherent spectroscopy. *J. Phys. Chem. Lett.* **9**, 5654–5659 (2018).
7. Kasha, M. Characterization of electronic transitions in complex molecules. *Discuss. Faraday Soc.* **9**, 14 (1950).
8. Perdomo-Ortiz, A., Widom, J. R., Lott, G. A., Aspuru-Guzik, A. & Marcus, A. H. Conformation and electronic population transfer in membrane-supported self-assembled porphyrin dimers by 2D fluorescence spectroscopy. *J. Phys. Chem. B* **116**, 10757–10770 (2012).
9. Mueller, S. *et al.* Fluorescence-detected two-quantum and one-quantum–two-quantum 2D electronic spectroscopy. *J. Phys. Chem. Lett.* **9**, 1964–1969 (2018).
10. Thyrhaug, E. *et al.* Identification and characterization of diverse coherences in the Fenna–Matthews–Olson complex. *Nat. Chem.* **10**, 780–786 (2018).
11. Christiansen, D. *et al.* Phonon sidebands in monolayer transition metal dichalcogenides. *Phys. Rev. Lett.* **119**, 187402 (2017).
12. Mukamel, S. *Principles of nonlinear optical spectroscopy*. (Oxford University Press, 1995).
13. Hao, K. *et al.* Neutral and charged inter-valley biexcitons in monolayer $MoSe_2$. *Nat. Commun.* **8**, 15552 (2017).
14. Li, Z. *et al.* Revealing the biexciton and trion-exciton complexes in BN encapsulated WSe_2. *Nat. Commun.* **9**, 3719 (2018).
15. Barbone, M. *et al.* Charge-tuneable biexciton complexes in monolayer WSe_2. *Nat. Commun.* **9**, 3721 (2018).

16. Qiu, D. Y., da Jornada, F. H. & Louie, S. G. Optical spectrum of MoS_2: Many-body effects and diversity of exciton states. *Phys. Rev. Lett.* **111**, 216805 (2013).

17. Chernikov, A. *et al.* Exciton binding energy and nonhydrogenic Rydberg series in monolayer WS_2. *Phys. Rev. Lett.* **113**, (2014).

18. Rethfeld, B., Ivanov, D. S., Garcia, M. E. & Anisimov, S. I. Modelling ultrafast laser ablation. *J. Phys. Appl. Phys.* **50**, 193001 (2017).

19. Wang, B. & Gallais, L. A theoretical investigation of the laser damage threshold of metal multi-dielectric mirrors for high power ultrashort applications. *Opt. Express* **21**, 14698–14711 (2013).

20. Yasaei, P. *et al.* Interfacial thermal transport in monolayer MoS_2- and Graphene-based devices. *Adv. Mater. Interfaces* **4**, 1700334 (2017).

21. Zhang, X. *et al.* Measurement of lateral and interfacial thermal conductivity of single- and bilayer MoS_2 and $MoSe_2$ using refined optothermal Raman technique. *ACS Appl. Mater. Interfaces* **7**, 25923–25929 (2015).

22. Yalon, E. *et al.* Energy dissipation in monolayer MoS_2 electronics. *Nano Lett.* **17**, 3429–3433 (2017).

23. Peng, B. *et al.* Thermal conductivity of monolayer MoS_2, $MoSe_2$, and WS_2: interplay of mass effect, interatomic bonding and anharmonicity. *RSC Adv.* **6**, 5767–5773 (2016).

24. Li, Y. *et al.* Measurement of the optical dielectric function of monolayer transition-metal dichalcogenides: MoS_2, $MoSe_2$, WS_2, and WSe_2. *Phys. Rev. B* **90**, 205422 (2014).
